# Health-based evaluation of ambient air measurements of PM_2.5_ and volatile organic compounds near a Marcellus Shale unconventional natural gas well pad site and a school campus

**DOI:** 10.1038/s41370-021-00298-5

**Published:** 2021-02-22

**Authors:** Christopher M. Long, Nicole L. Briggs, Brian A. Cochran, Destiny M. Mims

**Affiliations:** 1 Gradient, Boston, MA USA; 2 Gradient, Seattle, WA USA; 3 Spectrum Environmental Solutions, Austin, TX USA

**Keywords:** PM_2.5_, VOCs, Natural gas, Marcellus Shale, Air monitoring, Public health

## Abstract

**Background:**

Limited air monitoring studies with long-term measurements during all phases of development and production of natural gas and natural gas liquids have been conducted in close proximity to unconventional natural gas well pads.

**Objective:**

Conducted in an area of Washington County, Pennsylvania, with extensive Marcellus Shale development, this study investigated whether operations at an unconventional natural gas well pad may contribute to ambient air concentrations of potential health concern at a nearby school campus.

**Methods:**

Almost 2 years of air monitoring for fine particulate matter (PM_2.5_) and volatile organic compounds (VOCs) was performed at three locations between 1000 and 2800 feet from the study well pad from December 2016 to October 2018. PM_2.5_ was measured continuously at one of the three sites using a beta attenuation monitor, while 24-h stainless steel canister samples were collected every 6 days at all sites for analysis of 58 VOCs.

**Results:**

Mean PM_2.5_ concentrations measured during the different well activity periods ranged from 5.4 to 9.5 μg/m^3^, with similar levels and temporal changes as PM_2.5_ concentrations measured at a regional background location. The majority of VOCs were either detected infrequently or not at all, with measurements for a limited number of VOCs indicating the well pad to be a source of small and transient contributions.

**Significance:**

All measurement data of PM_2.5_ and 58 VOCs, which reflect the cumulative contributions of emissions from the study well pad and other local/regional air pollutant sources (e.g., other well pads), were below health-based air comparison values, and thus do not provide evidence of either 24-hour or long-term air quality impacts of potential health concern at the school.

## Introduction

There has been a proliferation of air monitoring data collected at major U.S. shale gas plays to understand the potential air quality impacts of the recent expansion of unconventional natural gas development (UNGD) activities, including horizontal drilling and hydraulic fracturing. Air measurement studies have been conducted by academic researchers [1–4], governmental agencies [5–9], industry scientists and industry-funded consultants [10–12], and environmental advocates and non-profit groups [13, 14]. Air pollutants that have been commonly measured in these studies include both US Environmental Protection Agency (US EPA) criteria air pollutants (e.g., fine particulate matter [PM_2.5_], nitrogen dioxide [NO_2_]), and volatile organic compounds (VOCs) classified by US EPA as air toxics (e.g., benzene, ethylbenzene, formaldehyde, n-hexane, toluene, and xylenes).

The Health Effects Institute (HEI)- Energy Research Committee [15] recently published a review of published air quality studies relevant to potential UNGD-related human exposures, identifying the need for additional studies to address important gaps in knowledge. In particular, the HEI-Energy report [15] highlighted the need for more research to characterize the spatial and temporal variability in airborne exposure levels and the conditions contributing to this variability, including more air monitoring data representing a range of geographic locales, meteorological conditions, UNGD operational conditions, and exposure durations (e.g., from acute durations of hours to weeks to chronic durations of a year and longer). In our review of air quality data available for the Marcellus Shale region [16], we observed that the majority of datasets consist of short-term measurements collected over time periods of days to weeks, thus providing insufficient data to evaluate long-term exposure conditions for the full life cycle of well pad development. In addition, most of the available measurement data are for monitoring locations between 0.2 and 1 miles from the nearest UNGD site, with fewer data for closer monitoring locations.

Pennsylvania’s Washington County is one area in the Marcellus Shale region that has experienced rapid unconventional natural gas development in the last 10–15 years. In Washington County alone, nearly 1700 unconventional wells have been drilled in the last decade, the most of any Pennsylvania county [17]. Public concerns have been raised regarding potential health risks posed by the proliferation of well pads and other associated natural gas infrastructure (e.g., compressor stations and processing facilities) in Washington County, with air emissions and exposures being particular concerns [7–9].

**Fig. 1:**
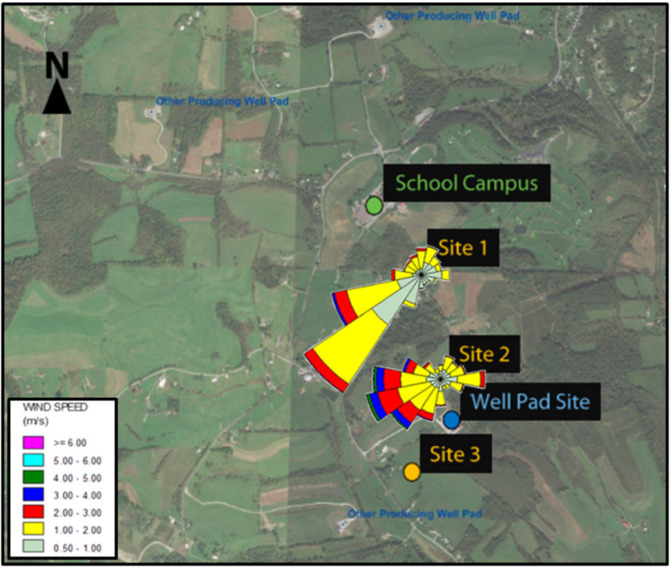
Map of the three air monitoring sites relative to the study well pad site, the school campus, and other local producing well pads. Wind roses are also shown for Sites 1 and 2 where meteorological stations were operated.

This air monitoring study was conducted in a part of Washington County with extensive Marcellus Shale development [18] (Figure S.1). The primary objective of the study was to investigate whether development activities and production operations at an unconventional natural gas well pad site may be contributing to ambient air concentrations of potential health concern at a nearby school campus. Almost 2 years of measurements for both PM_2.5_ and individual VOC species were made at three monitoring locations, including two locations between the well pad and the school campus and all between 1000 and 2800 feet from the well pad site (Fig. 1), during all phases of development and production of natural gas and natural gas liquids. Wind data (direction and speed) were also continuously collected at two of the monitoring sites. Thus, this dataset is notable for the lengthy duration of air quality measurements in close proximity to a well pad during all phases of development and production, and the collection of local wind data for assessing the contribution of the well pad to measured air concentrations.

We conducted a public health evaluation of this air monitoring dataset by comparing short-term (24-h) and long-term (>1 year) average PM_2.5_ and VOC concentrations to acute and chronic health-based air comparison values developed by public health agencies to serve as conservative and health-protective benchmarks. In addition, we compared PM_2.5_ and VOC measurements to air concentrations measured at a background Washington County site more distant from oil and gas development activities and considered to be representative of regional background air quality. Although the study was designed to identify potential air quality impacts at the nearby school campus associated with operations at the study well pad site, the collected dataset reflects the cumulative contributions of air emissions from both the study well pad site and other local and regional sources.

## Methods

### Ambient air measurements

Three air monitoring sites were selected to address the primary study objective of evaluating air quality impacts at a nearby school campus associated with the development and operation of a UNGD well pad. Air monitoring sites 1 and 2 were located at distances of ≈2800 and 1000 feet, respectively, from the study well pad in the direction of the school campus (Fig. 1). Site 1 was the closest to the school campus (≈1500 feet to the southeast). The third monitoring site (site 3) was located about 1000 feet to the southwest of the well pad —i.e., upwind of the well pad for winds blowing in the direction of the school— to help evaluate whether other local sources, including the large number of other UNGD wells in the area (Figure S.1), may be important contributors to the site 1 and 2 measurements. Although an initial evaluation of the wind direction in the area indicated that winds were predominantly from the southwest, a monitoring site was not established to the northeast of the well pad because the area is wooded and inaccessible.

The monitoring program began in December 2016 during the site construction and set-up period of the study well pad and continued through October 2018 and after a full year of measurements were collected with all wells (six in total) in production. Table 1 shows the study air monitoring period relative to the different well pad activity periods, which included each of the typical well pad development phases, periods of lesser activity between the development phases that we have termed interlude periods, and the period when all wells were in production.

**Table 1:** Sampling dates and numbers of PM_2.5_ measurement hours and VOC canister samples collected per study well pad activity period.

Study well pad activity period	Sampling dates	Number of monitoring site 1 PM_2.5_ measurement hours in the period	Percentage of total sampling hours	# of VOC canister samples for study monitoring sites		
				# 1	# 2	# 3
Site construction and set-up	December 16, 2016–January 5, 2017	N/A	N/A	0	4	4
Vertical air drilling	January 5–February 18, 2017	177	1.2%	0	7	7
Interlude I	February 19–March 2, 2017	240	1.7%	2	2	2
Horizontal drilling	March 3–May 7, 2017	1546	10.9%	11	11	11
Interlude II	May 8–June 17, 2017	964	6.8%	6	6	6
Hydraulic fracturing	June 18–August 13, 2017	1302	9.2%	10	10	10
Interlude III	August 14–September 7, 2017	439	3.1%	4	4	4
Flowback	September 8–October 23, 2017	883	6.2%	8	8	8
Production	October 23, 2017–October 31, 2018	8618	60.8%	61	61	61

*N/A* Not Applicable, *PM*_*2.5*_ fine particulate matter less than 2.5 micrometers in diameter, *VOC* volatile organic compound.

Ambient air measurements were made for PM_2.5_ and 58 VOC species (see Table 1 for numbers of PM_2.5_ measurement hours and VOC canister samples collected during each well pad activity period). Monitoring site 1 was chosen for the PM_2.5_ measurements given that it was between the study well pad site and the school campus and in closer proximity to the school campus than site 2. Hourly average PM_2.5_ measurements were collected continuously from February 2017 to October 2018 using a Met One Instruments Model BAM-1020 per the US EPA Federal Equivalent Method (FEM). Beginning on December 16, 2016, at sites 2 and 3, and February 15, 2018, at site 1, 24-h stainless steel canisters were collected for VOC analysis every 6 days through October 2018. Samples were analyzed using US EPA Method TO-15, focusing on 58 VOC species selected to match the set of TO-15 VOC species typically monitored by Pennsylvania Department of Environmental Protection (PADEP) at its air toxics sampling sites across the state. This expanded set of VOC analytes was selected based on prior experience of the well pad operator regarding typical air emission sources at its well pads. VOCs not known to be associated with UNGD activities (e.g., chlorinated solvents like carbon tetrachloride and methylene chloride) were retained as analytes. Acrolein was one of the 58 target VOCs, but we have not reported or evaluated the acrolein measurements based on determinations by both PADEP and US EPA that acrolein measurements obtained using this method are unreliable [8, 19, 20]. Wind speed and direction were also measured at both sites 1 and 2 using solar-powered portable met stations from February 8, 2017 to October 31, 2018, and December 16, 2016 to October 31, 2018, respectively; additional meteorological parameters (e.g., relative humidity, barometric pressure, and temperature) were also collected at site 1.

### Data analysis

Microsoft Excel 2013 (Microsoft Corporation, Redmond, WA, USA), SigmaPlot (Systat Software, Inc., San Jose, CA, USA), R (R Core Team, Vienna, Austria), and ProUCL version 5.1 (US EPA, Washington, DC, USA) were used for statistical and graphical data analysis. For PM_2.5_, we analyzed the hourly data, and also calculated 24-h daily average concentrations for days with 18 or more monitoring hours. For VOCs detected at least once, we substituted one-half the limit of detection (LOD) for non-detects (LODs were typically 0.06 parts per billion). The 95% upper confidence limits (UCLs) of mean concentrations were calculated for VOCs detected at least twice using US EPA’s ProUCL software, with reporting of UCLs for the methods recommended by the software.

Correlations between measured concentrations at each site were examined using Spearman rank correlations. We conducted statistical testing to compare concentrations between sites, well activity periods, and wind directions using non-parametric tests that included the Kruskal–Wallis *H* Test and the Mann–Whitney rank sum test. Statistical significance was defined as a *p* value less than 0.05. For VOCs, we focused statistical testing on a subset of 14 of the 58 target VOCs that were consistently detected (i.e., detection frequencies >75%) at each of the monitoring sites.

The wind direction data collected at sites 1 and 2 were evaluated in several ways. Wind roses were prepared using WRPLOT View (Lakes Environmental, Waterloo, Ontario). To allow for the evaluation of wind directions on a daily basis corresponding to the VOC sampling periods, average daily wind directions were calculated, categorized according to an 8-point compass, and the percent of days in which the winds arrived from each of these directions was calculated. Given the hourly averaging time of the PM_2.5_ measurements, the percent of hourly wind measurements in each of the eight directions was also calculated.

The PM_2.5_ and VOC measurements were also compared to air concentrations measured at a monitoring site ≈10 miles away in Florence, PA, which has been used by PADEP as a background Washington County comparison site [8]. PADEP has described this rural monitoring site as being impacted primarily by regional transport [8]. PM_2.5_ data for the study monitoring period were obtained for the Florence site from US EPA’s Air Quality System (AQS). No VOC data are available from the Florence site for the study monitoring period, but maximum 24-h and mean VOC concentrations for 24-h canister samples collected every sixth day between October 2012 and December 2013 at the Florence monitoring site were obtained from PADEP [8] data summaries.

### Health-based evaluation of ambient air measurements

We identified acute and chronic health-based air comparisons values (HBACVs) for this evaluation that are health-protective benchmarks developed by public health agencies. The US EPA PM_2.5_ primary National Ambient Air Quality Standards (NAAQS), which are developed to be protective of the health of the general public as well as sensitive populations such as asthmatics, children, and the elderly, were used as PM_2.5_ acute and chronic benchmarks. We compared the maximum 24-h daily average concentration to the level of the NAAQS (35 μg/m^3^), a conservative comparison given that the standard is intended to be compared to a 3-year average of the 98th percentile of 24-h measurements at a site. The annual PM_2.5_ NAAQS requires that the mean annual PM_2.5_ concentration at a site, averaged over 3 years, remains below 12.0 μg/m^3^. Given that PM_2.5_ measurements were not available for a 3-year period, the mean concentration from the entire PM_2.5_ sampling period was calculated and compared to the annual NAAQS.

The maximum 24-h measurement of each VOC detected at the three air monitoring sites was compared to acute HBACVs. We employed a tiered approach to identify acute HBACVs because there was not a single HBACV source inclusive of all measured VOCs. Agency for Toxic Substances and Disease Registry (ATSDR) acute inhalation Minimal Risk Levels (MRLs) were considered to be the preferred source of HBACVs because they are developed to be protective of 24-h exposure durations according to a well-documented and conservative process based on the most sensitive substance-induced end point of relevance to humans [21]. ATSDR acute inhalation MRLs are derived for 1–14 day exposure durations, and therefore comparison to the 24-h air monitoring site measurements is conservative. If an ATSDR acute inhalation MRL was not available for a VOC, acute inhalation reference concentrations (RfCs) from the Department of Energy Oak Ridge National Laboratory (ORNL) Risk Assessment Information System (RAIS) were used. When neither a ATSDR MRL nor a RAIS RfC was available, we derived an acute HBACV by multiplying a US EPA chronic reference concentration (RfC) by 10 [22]. For ethanol, the US National Institute for Occupational Safety and Health (NIOSH) time-weighted average recommended exposure limit (REL) was selected as the acute HBACV. We were not able to identify acute HBACVs for 11 VOCs, however, the majority of these were not detected in any samples.

We evaluated chronic health risks by comparing 95% UCLs of mean VOC concentrations (or for VOCs detected just once, mean concentrations that were calculated using half of the LOD for non-detects) at each site to chronic HBACVs. We consider 95% UCLs to represent conservative estimates of chronic air exposure levels at the monitoring sites given not only the likelihood that they are overestimates of true long-term average concentrations, but also due to the transient nature of the well pad development phases. For non-carcinogenic VOCs, US EPA non-cancer RfCs were used as chronic HBACVs, and for known or suspected human carcinogens, the lower value of either the non-cancer US EPA RfC or the cancer-based estimated continuous lifetime concentration was used. Using US EPA inhalation unit risk (IUR) estimates, we calculated the cancer-based estimated continuous lifetime concentrations for a 1-in-10,000 excess lifetime cancer risk, consistent with the US EPA residual risk program and with long-term comparison levels developed as part of US EPA’s School Air Toxics Initiative [22, 23].

## Results

### Wind measurement data

Wind roses constructed from wind data collected at monitoring sites 1 and 2 indicate that the prevailing local winds were from the west and southwest (Fig. 1), and thus did not generally blow emissions from the well pad towards the monitoring sites and the school campus. However, it is expected that winds blowing from the southerly and southeasterly directions would have transported study well pad air emissions to monitoring sites 1 and 2, and a detailed evaluation of wind directions at these sites confirmed that winds blowing from southeasterly and southerly directions were relatively common during each of the well pad activity periods (Table S.1).

### Summary of PM_2.5_ measurement data

**Fig. 2:**
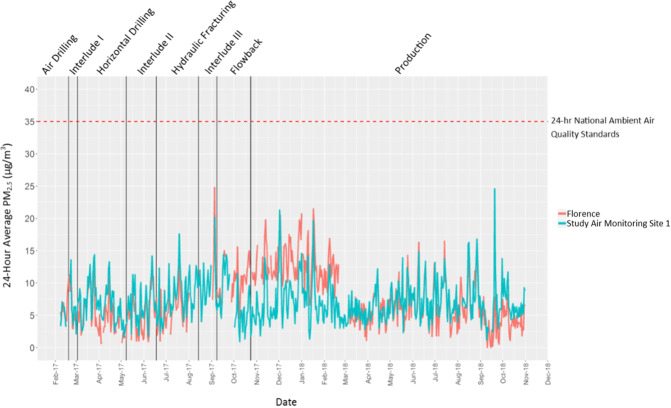
Time series of 24-h PM_2.5_ measurements at study monitoring site 1 and the PADEP background Florence site. PADEP Pennsylvania Department of Environmental Protection, PM_2.5_ fine particulate matter less than 2.5 micrometers in diameter.

Table 2 provides a summary of the hourly PM_2.5_ measurement data collected from February 2017 to October 2018 at monitoring site 1, showing an overall mean PM_2.5_ concentration of 7.1 μg/m^3^ and mean concentrations for the different well activity periods that ranged from a low of 5.4 μg/m^3^ for the vertical air drilling phase to a high of 9.5 μg/m^3^ for the interlude III phase. Kruskal–Wallis *H* Tests identified statistically significant differences in hourly PM_2.5_ concentrations between some of the well activity periods, including statistically higher concentrations for the interlude III and hydraulic fracturing periods and statistically lower concentrations for the vertical air drilling and production periods. When data were stratified by hours with winds from the south and southeast (i.e., from the direction of the study well pad site) versus winds from other directions, we observed statistically significant increased hourly PM_2.5_ concentrations for the hours with southerly and southeasterly wind directions for all well activity periods except the interlude II and interlude III periods (Figure S.2); however, as illustrated by Fig. 2 which compares 24-h average PM_2.5_ concentrations measured at monitoring site 1 with the corresponding 24-h average PM_2.5_ concentrations measured at the PADEP Florence background site, highly similar PM_2.5_ levels and temporal changes were observed as for a regional background site. Statistical testing showed no statistical difference between the two datasets (Mann–Whitney rank sum test, *p* value = 0.82).

**Table 2:** Summary of hourly PM_2.5_ measurements for monitoring site 1.

Study well pad activity period	Median hourly PM_2.5_ conc. (µg/m^3^)	Mean hourly PM_2.5_ conc. (µg/m^3^)	Standard deviation hourly PM_2.5_ conc. (µg/m^3^)	Maximum 1-h PM_2.5_ conc. (µg/m^3^)	Maximum 24-h PM_2.5_ conc. (µg/m^3^)
Site construction and set-up	N/A	N/A	N/A	N/A	N/A
Vertical air drilling	4.0	5.4	4.3	24.0	7.1
Interlude I	6.0	6.6	4.8	37.0	13.6
Horizontal drilling	6.0	6.8	4.4	54.0	14.4
Interlude II	6.0	6.8	4.0	29.0	14.2
Hydraulic fracturing	7.0	7.8	4.3	24.0	17.6
Interlude III	9.0	9.5	4.3	24.0	13.1
Flowback	6.0	7.0	4.9	41.0	14.4
Production	6.0	7.1	4.8	141.0	24.6
Total	6.0	7.1	4.7	141.0	24.6

The maximum 24-h PM_2.5_ concentration was calculated for only days in which there were at least 18 hours of PM_2.5_ data available.

N/A signifies that no data were collected for the period.

*Conc* concentration, *PM*_*2.5*_ fine particulate matter less than 2.5 micrometers in diameter

### Summary of VOC measurement data

Table S.2 provides a comprehensive set of summary statistics for the VOC measurement data by monitoring site, showing that the majority of the target VOC species were either detected infrequently or not at all. Only 14 VOCs were consistently detected (i.e., detection frequencies >75%) at each of the three monitoring sites—acetone, benzene, 2-butanone, carbon tetrachloride, chloromethane, dichlorodifluoromethane, ethanol, Freon 113, methanol, methylene chloride, n-hexane, propylene, toluene, and trichlorofluoromethane. While median concentrations for these VOCs were frequently less than 1 ppb and all were less than 10 ppb, maximum detected 24-h concentrations exceeded 100 ppb for a few of the VOCs (acetone, ethanol, and methanol). As shown in Table S.2, there were no consistent patterns with respect to when maximum VOC concentrations were detected across VOCs and monitoring sites. For example, maximum detected concentrations for both acetone and methanol occurred in the interlude II, production, and interlude III periods for monitoring sites 1, 2, and 3, respectively; for toluene, maximum detected concentrations occurred in the horizontal drilling, production, and interlude II periods for monitoring sites 1, 2, and 3, respectively. For a limited number of VOCs, maximum concentrations occurred within the same well activity period for either all three sites or two out of three sites (e.g., benzene: site construction and set-up period for two sites; n-hexane: flowback period for all three sites; ethanol: production period for two sites; propylene: flowback period for two sites).

Correlational analysis revealed consistent moderate to strong correlations (Spearman’s rank correlation coefficients r_s_ between 0.36 and 0.90) across the three monitoring sites between several groups of VOCs, suggesting that they may have common sources (Tables S. 3a, b, and c). These groupings included 2-butanone, acetone, ethanol, methanol, and toluene (for 2 of the 3 sites, also methylene chloride); chloromethane, dichlorodifluoromethane, and Freon 113; n-hexane and propylene (for 2 of the 3 sites, also toluene); and carbon tetrachloride and trichlorofluoromethane. Benzene exhibited statistically significant weak to moderate correlations (r_s_ between 0.31 and 0.44) with propylene and toluene for all sites and with n-hexane for 2 of the 3 sites. For site 1, we also examined correlations between 24-h daily-average PM_2.5_ concentrations and VOC concentrations, finding statistically significant weak correlations (r_s_ between 0.23 and 0.37) with benzene, carbon tetrachloride, methanol, n-hexane, and toluene. Although suggestive of possible common sources, the correlational analysis do not allow for the identification and apportionment of sources, such as any contributions from the study well pad site relative to other local well pads and area air emission sources (e.g., industrial sources and traffic).

Statistical testing using the Kruskal–Wallis *H* Test demonstrated no statistically significant differences in measured concentrations across the three monitoring sites for 9 of the 14 consistently detected VOCs. For the five VOCs where statistically significant differences by site were found (acetone, ethanol, methanol, methylene chloride, and toluene), multiple comparisons conducted using Dunn’s Method consistently showed statistically significantly higher concentrations at monitoring sites 2 and 3 versus monitoring site 1, but no statistically significant differences between the site 2 and site 3 concentrations.

Focusing on sites 1 and 2 where there were concurrent wind direction measurements, Tables S.4 and S.5 compare summary statistics for the 14 consistently detected VOCs for sampling days with frequent winds from the southerly or southeasterly direction (i.e., from the study well pad site in the direction of the monitoring sites and the school campus) versus for other wind directions. These tables show relatively small difference in concentrations for the two sets of wind conditions (i.e., typical <1 ppb differences in median concentrations). For a limited number of the 14 VOCs, statistically significant increased concentrations were observed for sampling days with frequent winds from south and southeasterly directions versus other wind directions, including for acetone (site 1), benzene (site 2), 2-butanone (site 2), ethanol (site 1), n-hexane (sites 1 and 2), propylene (site 2), and toluene (sites 1 and 2). However, for site 3 (which is to the southwest of the study well pad), most of the same VOCs (all but ethanol and 2-butanone) were found to have statistically significantly higher concentrations for days with frequent southerly and southeasterly winds versus other wind directions, suggesting that other local/regional sources rather than the study well pad site may be responsible for the higher concentrations at monitoring sites 1 and 2 with southerly and southeasterly winds (the wind data for monitoring site 2 were used in this analysis due to the lack of site-specific wind data for monitoring site 3).

Additional statistical testing was conducted on the VOC data to investigate whether measured VOC concentrations were related to study well pad activity period. Given the small number of samples for some of the shorter duration well activity periods, well development and interlude periods were grouped together to form three broader activity periods—active well development periods (encompassing the site construction and set-up, vertical air drilling, horizontal drilling, hydraulic fracturing, and flowback periods), interlude periods (encompassing the three interlude periods), and the production period. This statistical analysis identified some statistically significant differences in VOC concentrations for these activity periods, although the results were not consistent across VOCs and monitoring sites and are thus difficult to interpret. For example, no statistically significant differences across the three activity periods were observed in the Kruskal–Wallis *H* Test for benzene (*p* values of 0.562, 0.379, 0.086), ethanol (*p* values of 0.061, 0.551, 0.347), or n-hexane (*p* values of 0.396, 0.170, 0.464). However, for both methanol and propylene, statistically significant differences were observed for the activity period factor for each of the three sites, with pairwise multiple comparisons on ranks (Dunn’s Method) showing statistically significant lower methanol concentrations for the production period relative to the interlude periods for each site and to the active well pad development periods for one of the three sites, and statistically significant lower propylene concentrations for the production period relative to the active well pad development periods for all three sites.

Table S.6 compares summary statistics for VOCs measured at the three study monitoring sites with the corresponding values for 2012–2013 sampling conducted at the PADEP Florence background site [8]. As shown in this table, the same set of 12 VOCs was consistently detected at the Florence background site as at the study monitoring sites (note that neither ethanol nor methanol was monitored at the Florence site). Summary statistics were very similar between the two datasets for seven of the 12 VOCs, including benzene, 2-butanone, carbon tetrachloride, chloromethane, dichlorodifluoromethane, Freon 113, and trichlorofluoromethane. Six of these 7 VOCs are not well established to be associated with UNGD activities; although benzene is known to be present in UNGD site emissions, both mean and maximum benzene measurements for the study monitoring sites were generally lower than the Florence background site measurements. The 24-h maximum measurements for at least one of the three study monitoring sites were noticeably higher than the maximum measured Florence site concentrations for acetone, methylene chloride, n-hexane, propylene, and toluene. Although the study well pad site may have contributed to some of these maximum 24-h concentrations, an examination of the wind measurement data indicated that some of the maximum measurement days had few, if any, winds from the direction of the study well pad site, suggesting the role of other sources unrelated to the study well pad site.

### Comparison with health-based air comparison values (HBACVs)

The maximum 24-h PM_2.5_ concentration for the entire PM_2.5_ dataset was 24.6 μg/m^3^ (based on data from 587 days with at least 18 hours of PM_2.5_ data), which is well below the acute PM_2.5_ HBACV of 35 μg/m^3^. The overall mean PM_2.5_ concentration plus or minus one standard deviation was 7.1 ± 4.7 μg/m^3^, which is below the chronic PM_2.5_ HBACV of 12 μg/m^3^, even when including one standard deviation. Therefore, measured PM_2.5_ concentrations near the study well pad are below established regulatory levels of both acute and chronic health concern.

**Fig. 3:**
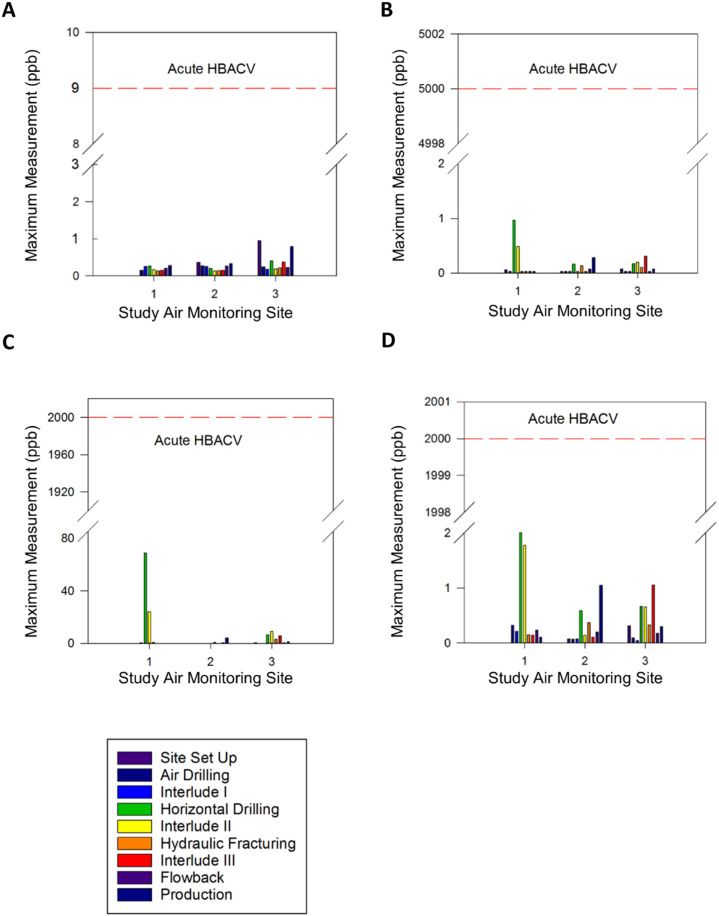
Summary of maximum measured 24-h VOC concentrations by monitoring site and study well pad site development phase. **A** benzene, **B** ethylbenzene, **C** toluene, and **D** xylenes. Acute health-based air comparison values (HBACVs) are shown in red dashed lines. ppb parts per billion, VOC volatile organic compound. Measurements for m-, p-, and o-xylenes are summed in this figure because the applicable acute HBACV is for mixed xylenes.

**Fig. 4:**
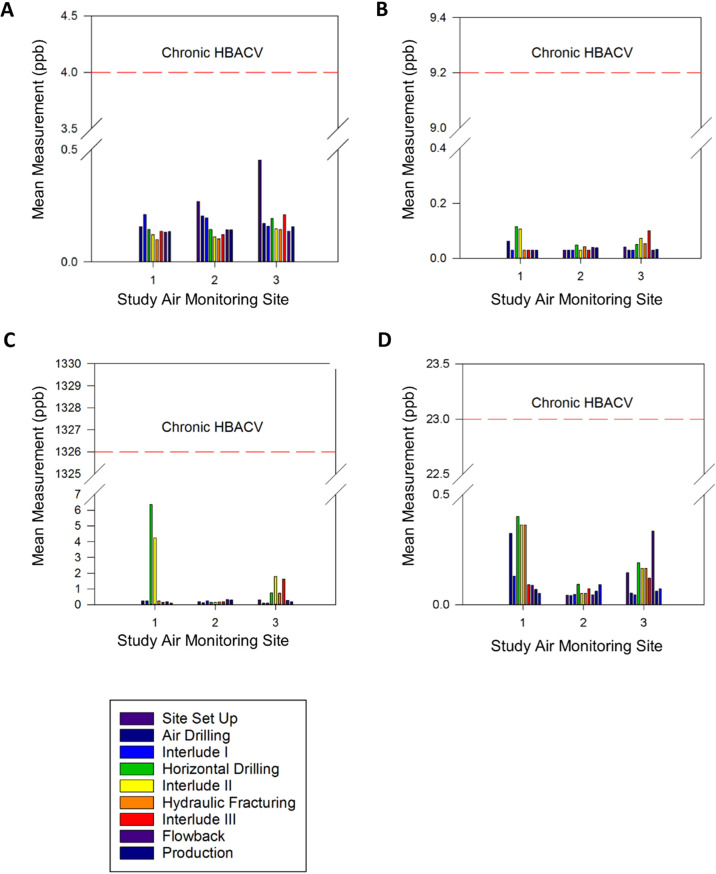
Summary of mean VOC concentrations by monitoring site and study well pad site development phase. **A** benzene, **B** ethylbenzene, **C** toluene, and **D** xylenes. Chronic health-based air comparison values (HBACVs) are shown in red dashed lines. ppb parts per billion, VOC volatile organic compound. Measurements for m-, p-, and o-xylenes are summed in this figure because the applicable chronic HBACV is for mixed xylenes. Mean concentrations, and not 95% UCLs of the means, are shown due to the small number of samples and high fraction of non-detects for some development phases.

For VOCs, Tables S.7 and S.8 provide the full set of comparisons to acute and chronic HBACVs. As shown in these tables, maximum measured 24-h VOC concentrations for each site were consistently below the acute HBACVs, while 95% UCLs of mean VOC concentrations calculated from all measurements and from only the production phase at each site were all below chronic HBACVs. Figures 3 and 4 illustrate the large differences that are typical between the measured VOC concentrations and the acute and chronic HBACVs for the BTEX compounds (benzene, toluene, ethylbenzene, and xylenes). There were four compounds detected at one or more of the air monitoring sites, but for which no appropriate acute or chronic benchmarks were identified: hexachloro-1,3-butadiene, m-dichlorobenzene, p-ethyltoluene, and trichlorofluoromethane. These VOCs are not expected to present either acute or chronic health risks due to the infrequent detections (for all but trichlorofluoromethane) and the low, sub-ppb detected concentrations (all).

## Discussion

Given the long duration of air monitoring, our study provided a dataset that reflects a range of well pad development phases and operating conditions, meteorological conditions, and exposure durations in the Marcellus Shale region. For PM_2.5_, the similar levels and diurnal trends between the study monitoring site and Florence background site indicate local/regional air quality as the dominant contributor to measured concentrations. Our analysis of PM_2.5_ measurements across the different well activity periods suggest possible small PM_2.5_ contributions at the measurement site from emissions at the study well pad site, such as for the hydraulic fracturing period; however, it bears mentioning that seasonal PM_2.5_ trends are a likely confounder for data comparisons between well activity periods, and our analysis of PM_2.5_ concentrations stratified by wind direction cannot differentiate between contributions from the study well pad and other local PM sources to the south and southeast. Both period-average and maximum 24-h concentrations for the well pad activity periods remained well below the US EPA NAAQS, indicating that if there were any PM_2.5_ air quality impacts from development activities at the study well pad site, they did not contribute to NAAQS exceedances at the monitoring site. Given the location of the monitoring site between the study well pad site and the school campus, it is thus unlikely that the study well pad site caused any PM_2.5_ NAAQS exceedances at the school campus.

Of the 14 consistently detected VOCs, seven (acetone, benzene, ethanol, methanol, n-hexane, propylene, toluene) have been associated with UNGD activities [9, 19, 24–26]. Of these seven VOCs, there were known sources of all but ethanol and methanol at the study well pad. Some of our study findings, including statistically significantly higher VOC concentrations (e.g., acetone, ethanol, methanol, methylene chloride, toluene) at the two monitoring sites closest to the study well pad site (sites 2 and 3) relative to the third site (1) and higher maximum 24-h VOC comparisons (e.g., acetone, methylene chloride, n-hexane, propylene, and toluene) at the study monitoring sites relative to data for the PADEP Florence background site, may indicate small and transient VOC contributions from the study well pad site at the monitoring sites. However, overall, there was significant variability in measured concentrations across different VOCs, sites, and sampling periods, and other findings suggest contributions from other local and regional sources. These findings include the measurement of maximum 24-h concentrations for a number of VOCs (e.g., acetone and methanol) during the nonactivity interlude periods at the study well pad site, and the similar statistically significant differences in VOC concentrations at monitoring site 3 with southerly and southeasterly winds as for monitoring sites 1 and 2. Half of the consistently detected VOCs (2-butanone, carbon tetrachloride, chloromethane, dichlorodifluoromethane, Freon 113, methylene chloride, trichlorofluoromethane) were frequently detected by PADEP during its short-term air monitoring studies conducted at UNGD sites in southwestern, northeastern, and northcentral PA, and attributed to either regional or global air quality rather than Marcellus Shale development activities [19, 24, 25]. Other target VOCs reported to be associated with well development activities (e.g., 1,3-butadiene, ethylbenzene, xylenes, trimethylbenzenes) were infrequently detected despite the use of sensitive detection limits.

Regardless of VOC sources, the measured concentrations, which reflect the cumulative contributions of both air emissions from the study well pad site and from other local and regional air pollutant sources including other area well pad sites, are consistently below levels of acute and chronic health concern. Given that two of the air monitoring sites are located between the study well pad site and the school campus, the VOC and PM_2.5_ measurement data do not provide evidence of either 24-h or long-term average concentrations of potential health concern at the nearby school campus. More study is needed to confirm their broader generalizability, but these study findings supporting the lack of elevated chronic exposure levels when PM_2.5_ and VOC concentrations were averaged across measurements made during all phases of well pad development may apply to other locales in the Marcellus Shale region with similar types of UNGD sites and operations.

These findings are consistent with operator efforts to control air emissions through continued refinement of best practices, as well as evolving governmental regulations focused on air emissions. Operators have made continuous improvements to improve drilling performance, completion design, and production efficiency [27]. For example, during drilling, VOC emission rates are kept relatively low since hydrocarbon zones have not been stimulated, and emissions are combusted as required for safety. Range Resources has developed an enhanced flowback process using updated equipment and processes that is estimated to reduce air emissions during flowback by more than 80% [27]. Design changes, including a transition from flare stacks and enclosed burner units to vapor recovery compression and closed-loop systems, and upgrades to thief hatches on tank batteries [27], have been implemented to eliminate episodic high emission rates. In addition, operators such as Range Resources have deployed advanced technologies, including supervisory control and data acquisition software, remote telemetry monitoring systems, and infrared optical methane cameras, in order to oversee production and quickly respond to potential problems [27]. As discussed in Seguljic and Martin [28], both federal and Pennsylvania state regulations have evolved in recent years to target air emissions from well pad development and production operations.

Other recent studies in the Marcellus Shale region have similarly reported measured air pollutant concentrations to be generally below levels of human health concern for air sampling conducted in proximity to UNGD sites [4, 8, 9, 16, 19, 24, 25, 29, 30]. In particular, the Maskrey et al. [30] study was conducted in the same community as this study to investigate air quality impacts of development activities at another local UNGD well pad at the same school campus. Conducted on behalf of the local school board, this study made continuous measurements of total volatile organic compound (TVOC) concentrations and collected canister samples for individual VOC analysis at two monitoring sites (on the high school campus and at a private residence) over an ≈3-month period during four well pad activity periods: a baseline period before hydraulic fracturing commenced, the hydraulic fracturing period, the flaring period, and an inactive period following flaring. None of the VOC concentrations measured at either the high school or the private residence exceeded health-based benchmarks, and therefore the study investigators concluded that there was no measurable health impact from the well pad at either site.

The Allegheny County Health Department (ACHD) collected one of the few other long-term datasets for the Marcellus Shale region that included monitoring during all phases of development at nearby well pads. ACHD installed the Deer Lakes and Imperial Pointe temporary monitors in 2014 ≈0.85 and 0.3 miles, respectively, from the nearest well pads. The 4 years of VOC data available for each of these sites prior to their decommissioning in May 2017 have been categorized by ACHD according to activity time periods (baseline, site construction, drilling, fracking, and production) at the nearest well pads [5, 6]. All measured VOC concentrations are consistently low and below health-based benchmarks; for example, the highest 24-h benzene concentration measured during the ACHD monitoring was 0.8 ppb, while study-average benzene concentrations of 0.17 and 0.26 ppb were measured at the two sites [16].

Some studies have reported findings of elevated episodic air pollutant concentrations near UNGD sites during specific phases of development [31, 32]. As part of the West Virginia University (WVU) Air, Noise, and Light Monitoring Study, McCawley [31] reported elevated maximum 72-h benzene concentrations ranging from 8.2 to 85 ppb at four UNGD sites during drilling (horizontal or vertical) or hydraulic fracturing/flowback activities. In comparison, maximum 24-h benzene concentrations for this study ranged from 0.29 to 0.95 ppb and were either lower than or only slightly above measured benzene concentrations for the PADEP Florence background site (Table S.6). Differences in these findings may be due in part to the closer proximity (between 492 and 1312 feet [33]) of the monitoring sites to well pads in the WVU study, as well as differences in well pad design and operations and processes. In addition, as mentioned previously, our study did not have a monitoring site in the prevailing wind direction, and it is thus not possible to rule out the presence of higher benzene (or other VOC) concentrations associated with the study well pad site at other non-monitored locations.

While we did not identify any clear, consistent patterns in short-term PM_2.5_ and VOC concentrations across the study well pad development phases, we acknowledge some important study limitations that have bearing on future studies investigating the temporal and spatial variability of air quality nearby to UNGD activity. Measurements in this study were focused on PM_2.5_ and VOCs, which are important classes of air pollutants that have been associated with UNGD. However, there are a number of other air pollutants that have also been associated with UNGD via primary emissions or secondary atmospheric formation, including other criteria air pollutants (NO_2_, carbon monoxide [CO], sulfur dioxide [SO_2_], ozone [O_3_]), and air toxics (e.g., acetaldehyde, formaldehyde, and hydrogen sulfide) [15, 16]. Hydrogen sulfide was not measured in this study based on prior analysis conducted by the site operator that indicated that this is not a sour gas region with significant hydrogen sulfide emissions. While it is thus not expected that hydrogen sulfide emissions at the well pad would have posed potential health risks at the school, this study did not address other air pollutants besides PM_2.5_ and VOCs.

Similar to other air monitoring studies, this study was limited by the small number of air monitoring sites, and for VOCs, by the 24-h sample averaging time and every 6th day sampling frequency. The PM_2.5_ and VOC measurement data provide estimates of air exposure levels at the monitoring sites themselves and may not be representative of other locations or time periods. For example, the limited number of air monitoring sites did not allow for the characterization of the full range of potential air exposure levels associated with the well pad development. However, for the primary study objective of evaluating air quality impacts of the study well pad at the school campus, the study design, and specifically the location of two of the monitoring sites between the study well pad site and the school campus, provided reliable evidence that air quality impacts of potential health concern were unlikely at the school. It is possible that higher VOC concentrations may have occurred on non-sampling days, however, it bears mentioning that the collection of 24-h samples every 6th day is the standard US EPA sampling design for air toxics [34]. Moreover, with greater than 1 year of air sampling during the production phase, there were more than 60 air samples collected for VOC analysis at each of the three air monitoring sites, and thus a sizable dataset to represent both 24-h peak and long-term average VOC concentrations during the production phase of the study well pad.

It is recommended that future air monitoring studies conducted in proximity to UNGD well pads include higher resolution sampling (e.g., 1-h) for VOCs, as the standard 24-h sample duration does not allow for the characterization of episodic peak air pollutant events. These data are needed to assess whether brief, intermittent exposures (i.e., 1-h or less) may pose acute health risks. Only a small number of studies conducted in the Marcellus Shale region have measured VOC concentrations for sampling frequencies of 1-h or less [1, 2, 31, 34, 35]. Given the difficulty of disentangling the contributions of a specific local well pad site from other area oil & gas development sites, it is also recommended that studies be designed to facilitate source apportionment modeling.

Our study results indicated some higher PM_2.5_ concentrations during the hydraulic fracturing phase when ≈100 temporary diesel-powered combustion sources (e.g., generators, light towers, pumps, pressure washers, heaters, and air compressors) are typically utilized at well pad sites. Although mean and maximum 24-h PM_2.5_ concentrations remained below the corresponding NAAQS during this time period, our findings indicate a need for additional PM_2.5_ monitoring during well completion activities to investigate possible off-site impacts of the combustion emissions. It bears mentioning that the industry has transitioned to greater direct use of natural gas in place of diesel fuel, or co-firing of natural gas with diesel fuel, for both drilling and well completion equipment [27, 36–38]. Flowback is generally recognized as a source of hydrocarbon emissions, and the high concentrations of some hydrocarbons (e.g., hexane and propylene) were detected during the flowback phase of the study well pad. Recognizing the recent transition to reduced emissions completions, our findings suggest that additional VOC monitoring during the flowback phase could be helpful to confirm the efficacy of reduced emissions completions for mitigating off-site VOC impacts.

In conclusion, this air quality and public health evaluation, which was designed to identify air quality impacts of potential health concern at a nearby school campus associated with operations of a Marcellus Shale unconventional gas well pad, showed that measured PM_2.5_ and VOC concentrations were consistently below acute and chronic health-based air comparison values. While the nearly 2 years of data collected at the three monitoring sites between 1000 and 2800 feet from the study well pad include some episodic short-term concentration increases that may be associated with the transient well pad development phases, the PM_2.5_ and VOC measurements do not provide evidence of elevated long-term average concentrations at the three monitoring sites relative to a Washington County background site more distant from Marcellus Shale development. The study measurement data, which reflect not only any air emissions from the study well pad but also air emissions from other local and regional Marcellus Shale development, do not provide evidence indicating that the study well pad was a source of either acute or chronic PM_2.5_ or VOC concentrations of potential health concern at the school campus; however, the study design did not include monitoring sites in the predominant wind direction or closer than 1000 feet from the well pad, and thus did not allow for the characterization of the full range of potential air exposure levels associated with the well pad development.

## Supplementary information

Supplementary Figures and Tables
