# Supplementary material for: Health-based evaluation of ambient air measurements of PM2.5 and volatile organic compounds near a Marcellus Shale unconventional natural gas well pad site and a school campus
Source: J Expo Sci Environ Epidemiol. 2021 Feb 22;31(4):614–27. doi: 10.1038/s41370-021-00298-5 (PMC8263344; doi:10.1038/s41370-021-00298-5)
Supplement: Supplementary file 1 — Supplementary Figures and Tables [file 41370_2021_298_MOESM1_ESM.docx]

**Table S.1 Wind Direction Evaluation for Monitoring Sites 1 and 2**

| **Monitoring Site 1** | | | | | | | | | | | | | | | | | |
| --- | --- | --- | --- | --- | --- | --- | --- | --- | --- | --- | --- | --- | --- | --- | --- | --- | --- |
| **Study Well Pad Activity Period** | **Average Wind Speed (mph)** | **Percent of Days with Average Wind Direction in Category (%):** | | | | | | | | **Percent of Hours with Wind Direction in Category (%):** | | | | | | | |
|  |  | **N** | **NE** | **E** | **SE** | **S** | **SW** | **W** | **NW** | **N** | **NE** | **E** | **SE** | **S** | **SW** | **W** | **NW** |
| Total | 4 | 0 | 5 | 10 | 10 | 24 | 38 | 12 | 1 | 9 | 9 | 10 | 7 | 8 | 36 | 14 | 6 |
| Site Set-up (Before Jan 5, 2017) | N/A | N/A | N/A | N/A | N/A | N/A | N/A | N/A | N/A | N/A | N/A | N/A | N/A | N/A | N/A | N/A | N/A |
| Air Drilling (Jan 5, 2017 – Feb 18, 2017) | 6 | 0 | 9 | 0 | 0 | 9 | 55 | 27 | 0 | 5 | 2 | 2 | 6 | 5 | 41 | 31 | 8 |
| Interlude I (Feb 19, 2017 – Mar 2, 2017) | 9 | 0 | 0 | 0 | 0 | 33 | 42 | 25 | 0 | 1 | 1 | 7 | 3 | 7 | 53 | 26 | 1 |
| Horizontal Drilling (Mar 3, 2017 – May 7, 2017) | 5 | 0 | 2 | 18 | 8 | 20 | 30 | 23 | 0 | 10 | 6 | 18 | 6 | 4 | 29 | 18 | 9 |
| Interlude II (May 8, 2017 – Jun 17, 2017) | 3 | 0 | 2 | 15 | 5 | 22 | 54 | 0 | 2 | 6 | 10 | 12 | 5 | 9 | 38 | 14 | 7 |
| Hydraulic Fracturing (Jun 18, 2017 – Aug 13, 2017) | 3 | 0 | 4 | 7 | 9 | 25 | 47 | 9 | 0 | 10 | 7 | 4 | 8 | 11 | 46 | 11 | 5 |
| Interlude III (Aug 14, 2017 – Sep 7, 2017) | 2 | 0 | 8 | 16 | 8 | 20 | 44 | 4 | 0 | 8 | 14 | 10 | 9 | 13 | 31 | 11 | 4 |
| Flowback (Sep 8, 2017 – Oct 23, 2017) | 2 | 0 | 7 | 11 | 24 | 37 | 20 | 2 | 0 | 10 | 11 | 18 | 13 | 13 | 27 | 5 | 3 |
| Production (After Oct 23, 2017) | 4 | 0 | 6 | 9 | 10 | 24 | 38 | 12 | 1 | 10 | 9 | 9 | 7 | 8 | 36 | 14 | 6 |

| **Monitoring Site 2** | | | | | | | | | | | | | | | | | |
| --- | --- | --- | --- | --- | --- | --- | --- | --- | --- | --- | --- | --- | --- | --- | --- | --- | --- |
| **Study Well Pad Activity Period** | **Average Wind Speed (mph)** | **Percent of Days with Average Wind Direction in Category (%):** | | | | | | | | **Percent of Hours with Wind Direction in Category (%):** | | | | | | | |
|  |  | **N** | **NE** | **E** | **SE** | **S** | **SW** | **W** | **NW** | **N** | **NE** | **E** | **SE** | **S** | **SW** | **W** | **NW** |
| Total | 5 | 0 | 3 | 14 | 12 | 21 | 31 | 17 | 2 | 6 | 8 | 13 | 5 | 9 | 20 | 22 | 7 |
| Site Set-up (Before Jan 5, 2017) | 9 | 0 | 0 | 6 | 6 | 25 | 31 | 31 | 0 | 1 | 2 | 11 | 13 | 8 | 21 | 40 | 4 |
| Air Drilling (Jan 5, 2017 – Feb 18, 2017) | 8 | 0 | 0 | 6 | 0 | 35 | 12 | 47 | 0 | 4 | 5 | 6 | 7 | 8 | 19 | 39 | 12 |
| Interlude I (Feb 19, 2017 – Mar 2, 2017) | 9 | 0 | 0 | 0 | 0 | 42 | 25 | 33 | 0 | 0 | 0 | 3 | 9 | 14 | 32 | 38 | 4 |
| Horizontal Drilling (Mar 3, 2017 – May 7, 2017) | 8 | 0 | 5 | 17 | 8 | 17 | 31 | 20 | 3 | 8 | 8 | 18 | 6 | 8 | 15 | 24 | 12 |
| Interlude II (May 8, 2017 – Jun 17, 2017) | 5 | 0 | 2 | 12 | 7 | 22 | 44 | 10 | 2 | 4 | 9 | 15 | 4 | 7 | 27 | 27 | 7 |
| Hydraulic Fracturing (Jun 18, 2017 – Aug 13, 2017) | 6 | 0 | 2 | 7 | 7 | 12 | 44 | 28 | 0 | 6 | 9 | 5 | 3 | 5 | 32 | 33 | 7 |
| Interlude III (Aug 14, 2017 – Sep 7, 2017) | 5 | 0 | 4 | 20 | 16 | 12 | 32 | 16 | 0 | 5 | 11 | 22 | 5 | 5 | 19 | 27 | 6 |
| Flowback (Sep 8, 2017 – Oct 23, 2017) | 4 | 0 | 2 | 13 | 30 | 30 | 17 | 7 | 0 | 8 | 12 | 26 | 7 | 9 | 18 | 14 | 7 |
| Production (After Oct 23, 2017) | 4 | 0 | 3 | 16 | 13 | 22 | 30 | 13 | 3 | 7 | 8 | 13 | 5 | 11 | 20 | 19 | 7 |

Notes:

N/A signifies that no data were available for the period.

**Table S.2 Overall VOC Summary Statistics by Study Monitoring Site**

| **Compound** | **Monitoring Site 1** | | | | | | | | **Monitoring Site 2** | | | | | | | | **Monitoring Site 3** | | | | | | | |
| --- | --- | --- | --- | --- | --- | --- | --- | --- | --- | --- | --- | --- | --- | --- | --- | --- | --- | --- | --- | --- | --- | --- | --- | --- |
|  | **No. of Meas.** | **No. of Det.** | **% Det. Freq.** | **Mean Conc. (ppb)^1^** | **Median Conc. (ppb)^1^** | **95% UCLM (ppb)** | **Max Conc. (ppb)** | **Study Well Pad Activity Period in Which Max Conc. Occurred** | **No. of Meas.** | **No. of Det.** | **% Det. Freq.** | **Mean Conc. (ppb)^1^** | **Median Conc. (ppb)^1^** | **95% UCLM (ppb)** | **Max Conc. (ppb)** | **Study Well Pad Activity Period in Which Max Conc. Occurred** | **No. of Meas.** | **No. of Det.** | **% Det. Freq.** | **Mean Conc. (ppb)^1^** | **Median Conc. (ppb)^1^** | **95% UCLM (ppb)** | **Max Conc. (ppb)** | **Study Well Pad Activity Period in Which Max Conc. Occurred** |
| 1,1,1-Trichloro-ethane | 103 | 0 | 0% |  |  |  |  |  | 113 | 0 | 0% |  |  |  |  |  | 113 | 0 | 0% |  |  |  |  |  |
| 1,1,2,2-Tetra-chloro-ethane | 103 | 0 | 0% |  |  |  |  |  | 113 | 0 | 0% |  |  |  |  |  | 113 | 0 | 0% |  |  |  |  |  |
| 1,1,2-Trichloro-ethane | 103 | 1 | 1% | 0.03 | 0.03 |  | 0.07 | Production | 113 | 0 | 0% |  |  |  |  |  | 113 | 1 | 1% | 0.03 | 0.03 |  | 0.12 | Interlude III |
| 1,1-Dichloro-ethane | 103 | 0 | 0% |  |  |  |  |  | 113 | 0 | 0% |  |  |  |  |  | 113 | 0 | 0% |  |  |  |  |  |
| 1,1-Dichloro-ethylene | 103 | 0 | 0% |  |  |  |  |  | 113 | 0 | 0% |  |  |  |  |  | 113 | 0 | 0% |  |  |  |  |  |
| 1,2,4-Trichloro-benzene | 103 | 2 | 2% | 0.03 | 0.03 | 0.06 | 0.15 | Production | 113 | 3 | 3% | 0.03 | 0.03 | 0.06 | 0.10 | Hydraulic Fracturing | 113 | 3 | 3% | 0.03 | 0.03 | 0.06 | 0.12 | Horizontal Drilling |
| 1,2,4-Trimethyl-benzene | 103 | 7 | 7% | 0.08 | 0.03 | 0.30 | 2.71 | Horizontal Drilling | 113 | 12 | 11% | 0.04 | 0.03 | 0.08 | 0.40 | Horizontal Drilling | 113 | 8 | 7% | 0.04 | 0.03 | 0.08 | 0.46 | Horizontal Drilling |
| 1,2-Dibromo-ethane | 103 | 0 | 0% |  |  |  |  |  | 113 | 0 | 0% |  |  |  |  |  | 113 | 0 | 0% |  |  |  |  |  |
| 1,2-Dichloro-ethane | 103 | 3 | 3% | 0.03 | 0.03 | 0.06 | 0.12 | Horizontal Drilling | 113 | 3 | 3% | 0.03 | 0.03 | 0.06 | 0.17 | Production | 113 | 5 | 4% | 0.04 | 0.03 | 0.08 | 0.57 | Interlude III |
| 1,2-Dichloro-propane | 103 | 0 | 0% |  |  |  |  |  | 113 | 0 | 0% |  |  |  |  |  | 113 | 1 | 1% | 0.03 | 0.03 |  | 0.12 | Interlude III |
| 1,3,5-Trimethyl-benzene | 103 | 3 | 3% | 0.04 | 0.03 | 0.08 | 0.79 | Horizontal Drilling | 113 | 5 | 4% | 0.03 | 0.03 | 0.06 | 0.14 | Hydraulic Fracturing | 113 | 3 | 3% | 0.03 | 0.03 | 0.07 | 0.24 | Horizontal Drilling |
| 1,3-Butadiene | 103 | 2 | 2% | 0.03 | 0.03 | 0.06 | 0.06 | Horizontal Drilling | 113 | 1 | 1% | 0.03 | 0.03 |  | 0.12 | Interlude II | 113 | 8 | 7% | 0.04 | 0.03 | 0.07 | 0.26 | Site Construction and Set-up |
| 1-Bromo-propane | 103 | 1 | 1% | 0.03 | 0.03 |  | 0.25 | Production | 113 | 1 | 1% | 0.03 | 0.03 |  | 0.07 | Production | 113 | 0 | 0% |  |  |  |  |  |
| 2-Butanone | 103 | 99 | 96% | 0.45 | 0.33 | 0.51 | 1.93 | Production | 113 | 111 | 98% | 0.54 | 0.42 | 0.60 | 2.08 | Horizontal Drilling | 113 | 110 | 97% | 0.61 | 0.38 | 0.68 | 5.77 | Hydraulic Fracturing |
| 2-Hexanone | 103 | 17 | 17% | 0.04 | 0.03 | 0.07 | 0.14 | Hydraulic Fracturing | 113 | 25 | 22% | 0.05 | 0.03 | 0.08 | 0.27 | Hydraulic Fracturing | 113 | 26 | 23% | 0.06 | 0.03 | 0.13 | 1.27 | Hydraulic Fracturing |
| Acetone | 103 | 99 | 96% | 6.01 | 2.88 | 5.13 | 212.00 | Interlude II | 113 | 111 | 98% | 5.40 | 4.23 | 6.09 | 27.70 | Production | 113 | 110 | 97% | 5.86 | 3.39 | 6.69 | 43.50 | Interlude III |
| Benzene | 103 | 95 | 92% | 0.13 | 0.12 | 0.15 | 0.29 | Production | 113 | 109 | 96% | 0.15 | 0.13 | 0.15 | 0.37 | Site Construction and Set-up | 113 | 108 | 96% | 0.17 | 0.14 | 0.17 | 0.95 | Site Construction and Set-up |
| Bromo-dichloro-methane | 103 | 0 | 0% |  |  |  |  |  | 113 | 0 | 0% |  |  |  |  |  | 113 | 0 | 0% |  |  |  |  |  |
| Bromo-form | 103 | 0 | 0% |  |  |  |  |  | 113 | 0 | 0% |  |  |  |  |  | 113 | 0 | 0% |  |  |  |  |  |
| Bromo-methane | 103 | 0 | 0% |  |  |  |  |  | 113 | 1 | 1% | 0.03 | 0.03 |  | 0.07 | Production | 113 | 1 | 1% | 0.03 | 0.03 |  | 0.07 | Interlude II |
| c-1,2-Dichloro-ethylene | 103 | 0 | 0% |  |  |  |  |  | 113 | 0 | 0% |  |  |  |  |  | 113 | 0 | 0% |  |  |  |  |  |
| c-1,3-Dichloro-propene | 103 | 0 | 0% |  |  |  |  |  | 113 | 0 | 0% |  |  |  |  |  | 113 | 0 | 0% |  |  |  |  |  |
| Carbon Tetra-chloride | 103 | 99 | 96% | 0.08 | 0.08 | 0.09 | 0.11 | Production | 113 | 109 | 96% | 0.09 | 0.08 | 0.09 | 0.21 | Production | 113 | 110 | 97% | 0.08 | 0.08 | 0.09 | 0.13 | Production |
| Chloro-benzene | 103 | 0 | 0% |  |  |  |  |  | 113 | 2 | 2% | 0.03 | 0.03 | 0.06 | 0.06 | Hydraulic Fracturing | 113 | 1 | 1% | 0.03 | 0.03 |  | 0.07 | Horizontal Drilling |
| Chloro-ethane | 103 | 9 | 9% | 0.04 | 0.03 | 0.07 | 0.21 | Interlude II | 113 | 12 | 11% | 0.04 | 0.03 | 0.07 | 0.55 | Interlude II | 113 | 11 | 10% | 0.06 | 0.03 | 0.08 | 1.37 | Hydraulic Fracturing |
| Chloroform | 103 | 3 | 3% | 0.03 | 0.03 | 0.07 | 0.25 | Interlude II | 113 | 4 | 4% | 0.07 | 0.03 | 0.15 | 3.21 | Interlude II | 113 | 4 | 4% | 0.04 | 0.03 | 0.08 | 0.57 | Horizontal Drilling |
| Chloro-methane | 103 | 99 | 96% | 0.56 | 0.54 | 0.59 | 1.62 | Interlude II | 113 | 111 | 98% | 0.57 | 0.54 | 0.61 | 2.21 | Interlude II | 113 | 110 | 97% | 0.56 | 0.52 | 0.60 | 1.66 | Interlude II |
| Cyclo-hexane | 103 | 26 | 25% | 0.06 | 0.03 | 0.13 | 1.10 | Horizontal Drilling | 113 | 34 | 30% | 0.08 | 0.03 | 0.20 | 2.49 | Production | 113 | 43 | 38% | 0.08 | 0.03 | 0.16 | 0.89 | Flowback |
| Dibromo-chloro-methane | 103 | 0 | 0% |  |  |  |  |  | 113 | 0 | 0% |  |  |  |  |  | 113 | 0 | 0% |  |  |  |  |  |
| Dichloro-difluoro-methane | 103 | 99 | 96% | 0.41 | 0.44 | 0.43 | 0.63 | Production | 113 | 110 | 97% | 0.43 | 0.44 | 0.45 | 0.93 | Production | 113 | 109 | 96% | 0.42 | 0.44 | 0.44 | 0.68 | Production |
| Ethanol | 103 | 95 | 92% | 2.19 | 1.81 | 2.94 | 9.86 | Horizontal Drilling | 113 | 108 | 96% | 16.27 | 2.81 | 36.17 | 442.00 | Production | 113 | 106 | 94% | 73.42 | 2.26 | 308.20 | 5900.00 | Production |
| Ethyl-benzene | 103 | 3 | 3% | 0.04 | 0.03 | 0.09 | 0.97 | Horizontal Drilling | 113 | 9 | 8% | 0.04 | 0.03 | 0.07 | 0.29 | Production | 113 | 14 | 12% | 0.04 | 0.03 | 0.08 | 0.31 | Interlude III |
| Freon 113 | 103 | 92 | 89% | 0.07 | 0.07 | 0.07 | 0.09 | Hydraulic Fracturing | 113 | 105 | 93% | 0.07 | 0.07 | 0.08 | 0.14 | Production | 113 | 103 | 91% | 0.07 | 0.07 | 0.07 | 0.10 | Interlude II |
| Freon 114 | 103 | 0 | 0% |  |  |  |  |  | 113 | 0 | 0% |  |  |  |  |  | 113 | 0 | 0% |  |  |  |  |  |
| Hexachloro-1,3-Butadiene | 103 | 1 | 1% | 0.03 | 0.03 |  | 0.06 | Horizontal Drilling | 113 | 0 | 0% |  |  |  |  |  | 113 | 1 | 1% | 0.03 | 0.03 |  | 0.08 | Horizontal Drilling |
| m-Dichloro-benzene | 103 | 3 | 3% | 0.03 | 0.03 | 0.06 | 0.09 | Production | 113 | 3 | 3% | 0.03 | 0.03 | 0.06 | 0.15 | Hydraulic Fracturing | 113 | 2 | 2% | 0.03 | 0.03 | 0.07 | 0.17 | Horizontal Drilling |
| Methanol | 103 | 99 | 96% | 8.44 | 4.97 | 13.27 | 82.20 | Interlude II | 113 | 111 | 98% | 11.08 | 7.67 | 12.93 | 54.80 | Production | 113 | 110 | 97% | 13.29 | 6.97 | 15.26 | 122.00 | Interlude III |
| Methyl t-Butylether | 103 | 0 | 0% |  |  |  |  |  | 113 | 0 | 0% |  |  |  |  |  | 113 | 1 | 1% | 0.03 | 0.03 |  | 0.13 | Hydraulic Fracturing |
| Methylene Chloride | 103 | 86 | 83% | 0.72 | 0.11 | 2.52 | 30.30 | Flowback | 113 | 98 | 87% | 1.15 | 0.21 | 2.39 | 27.40 | Flowback | 113 | 103 | 91% | 1.00 | 0.18 | 2.08 | 19.70 | Interlude II |
| Methyl-isobutyl-ketone | 103 | 4 | 4% | 0.03 | 0.03 | 0.07 | 0.18 | Interlude II | 113 | 12 | 11% | 0.04 | 0.03 | 0.07 | 0.12 | Production | 113 | 12 | 11% | 0.04 | 0.03 | 0.08 | 0.31 | Hydraulic Fracturing |
| n-Heptane | 103 | 63 | 61% | 0.11 | 0.08 | 0.19 | 1.03 | Horizontal Drilling | 113 | 76 | 67% | 0.12 | 0.08 | 0.20 | 1.32 | Flowback | 113 | 80 | 71% | 0.14 | 0.09 | 0.26 | 2.47 | Flowback |
| n-Hexane | 103 | 96 | 93% | 0.23 | 0.14 | 0.38 | 2.37 | Flowback | 113 | 101 | 89% | 0.27 | 0.17 | 0.47 | 4.28 | Flowback | 113 | 102 | 90% | 0.31 | 0.17 | 0.29 | 7.57 | Flowback |
| o-Dichloro-benzene | 103 | 2 | 2% | 0.03 | 0.03 | 0.06 | 0.07 | Production | 113 | 2 | 2% | 0.03 | 0.03 | 0.06 | 0.09 | Hydraulic Fracturing | 113 | 2 | 2% | 0.03 | 0.03 | 0.07 | 0.16 | Horizontal Drilling |
| o-Xylene | 103 | 6 | 6% | 0.05 | 0.03 | 0.07 | 1.14 | Horizontal Drilling | 113 | 12 | 11% | 0.04 | 0.03 | 0.08 | 0.33 | Production | 113 | 13 | 12% | 0.04 | 0.03 | 0.07 | 0.32 | Interlude III |
| p-Dichloro-benzene | 103 | 2 | 2% | 0.03 | 0.03 | 0.06 | 0.13 | Production | 113 | 2 | 2% | 0.03 | 0.03 | 0.06 | 0.15 | Hydraulic Fracturing | 113 | 2 | 2% | 0.03 | 0.03 | 0.06 | 0.16 | Horizontal Drilling |
| p-Ethyl-toluene | 103 | 3 | 3% | 0.04 | 0.03 | 0.08 | 0.55 | Horizontal Drilling | 113 | 5 | 4% | 0.04 | 0.03 | 0.07 | 0.26 | Hydraulic Fracturing | 113 | 3 | 3% | 0.03 | 0.03 | 0.07 | 0.29 | Horizontal Drilling |
| Propylene | 103 | 98 | 95% | 1.21 | 0.61 | 2.22 | 14.30 | Interlude II | 113 | 108 | 96% | 1.12 | 0.66 | 1.97 | 18.00 | Flowback | 113 | 108 | 96% | 1.33 | 0.68 | 1.30 | 32.40 | Flowback |
| p-Xylene + m-Xylene | 103 | 26 | 25% | 0.09 | 0.05 | 0.23 | 2.40 | Horizontal Drilling | 113 | 35 | 31% | 0.08 | 0.05 | 0.14 | 0.72 | Production | 113 | 40 | 35% | 0.08 | 0.05 | 0.14 | 0.74 | Interlude III |
| Styrene | 103 | 2 | 2% | 0.04 | 0.03 | 0.09 | 0.48 | Horizontal Drilling | 113 | 26 | 23% | 0.07 | 0.03 | 0.11 | 0.73 | Production | 113 | 22 | 19% | 0.06 | 0.03 | 0.12 | 0.65 | Interlude II |
| t-1,2-Dichloro-ethylene | 103 | 0 | 0% |  |  |  |  |  | 113 | 0 | 0% |  |  |  |  |  | 113 | 0 | 0% |  |  |  |  |  |
| t-1,3-Dichloro-propene | 103 | 0 | 0% |  |  |  |  |  | 113 | 0 | 0% |  |  |  |  |  | 113 | 0 | 0% |  |  |  |  |  |
| Tetra-chloro-ethylene | 103 | 2 | 2% | 0.03 | 0.03 | 0.06 | 0.13 | Horizontal Drilling | 113 | 5 | 4% | 0.03 | 0.03 | 0.06 | 0.21 | Production | 113 | 1 | 1% | 0.03 | 0.03 |  | 0.07 | Horizontal Drilling |
| Tetra-hydrofuran | 103 | 9 | 9% | 0.04 | 0.03 | 0.07 | 0.30 | Horizontal Drilling | 113 | 14 | 12% | 0.04 | 0.03 | 0.09 | 0.50 | Vertical Air Drilling | 113 | 16 | 14% | 0.04 | 0.03 | 0.07 | 0.40 | Site Construction and Set-up |
| Toluene | 103 | 95 | 92% | 1.08 | 0.11 | 4.27 | 68.70 | Horizontal Drilling | 113 | 102 | 90% | 0.25 | 0.15 | 0.43 | 4.27 | Production | 113 | 102 | 90% | 0.43 | 0.17 | 0.94 | 9.16 | Interlude II |
| Trichloro-ethylene | 103 | 1 | 1% | 0.03 | 0.03 |  | 0.07 | Horizontal Drilling | 113 | 1 | 1% | 0.03 | 0.03 |  | 0.11 | Vertical Air Drilling | 113 | 0 | 0% |  |  |  |  |  |
| Trichloro-fluoro-methane | 103 | 99 | 96% | 0.24 | 0.24 | 0.25 | 0.33 | Hydraulic Fracturing | 113 | 111 | 98% | 0.26 | 0.24 | 0.27 | 0.58 | Production | 113 | 110 | 97% | 0.25 | 0.24 | 0.25 | 0.38 | Flowback |
| Vinyl Chloride | 103 | 0 | 0% |  |  |  |  |  | 113 | 1 | 1% | 0.03 | 0.03 |  | 0.07 | Interlude II | 113 | 0 | 0% |  |  |  |  |  |
| Xylenes | 103 | 27 | 26% | 0.12 | 0.05 | 0.32 | 3.54 | Horizontal Drilling | 113 | 35 | 31% | 0.10 | 0.05 | 0.19 | 1.05 | Production | 113 | 41 | 36% | 0.10 | 0.05 | 0.19 | 1.06 | Interlude III |

Notes:

Conc. = Concentration; Det. = Detection; Freq. = Frequency; Meas. = Measurement; No. = Number; ppb = Parts Per Billion; SD = Standard Deviation.

(1) Half of the detection limit was substituted for non-detects.

**Table S.3a Summary of Spearman Rank Correlation Results for 24-hour Measurement Data by Monitoring Site – Site 1**

|  | **Acetone** | **Benzene** | **Carbon  Tetrachloride** | **Chloromethane** | **Dichloro- difluoromethane** | **Ethanol** | **Freon 113** | **Methanol** | **Methylene  Chloride** | **n-Hexane** | **Propylene** | **Toluene** | **Trichloro- fluoromethane** | **24-hr PM_2.5_** |
| --- | --- | --- | --- | --- | --- | --- | --- | --- | --- | --- | --- | --- | --- | --- |
| 2-Butanone | 0.896 | -0.234 | 0.0279 | 0.266 | 0.0497 | 0.557 | 0.0824 | 0.733 | 0.266 | 0.429 | 0.321 | 0.405 | 0.0227 | 0.132 |
|  | 0.0000002 | 0.0198 | 0.784 | 0.00783 | 0.625 | 0.00000000172 | 0.417 | 0.0000002 | 0.00794 | 0.0000109 | 0.00127 | 0.000036 | 0.823 | 0.191 |
|  | 99 | 99 | 99 | 99 | 99 | 99 | 99 | 99 | 99 | 99 | 99 | 99 | 99 | 99 |
| Acetone |  | -0.305 | 0.0839 | 0.293 | 0.0645 | 0.618 | 0.068 | 0.77 | 0.22 | 0.465 | 0.244 | 0.465 | 0.0479 | 0.132 |
|  |  | 0.00225 | 0.408 | 0.0033 | 0.525 | 0.0000002 | 0.503 | 0.0000002 | 0.029 | 0.00000155 | 0.0152 | 0.00000151 | 0.637 | 0.192 |
|  |  | 99 | 99 | 99 | 99 | 99 | 99 | 99 | 99 | 99 | 99 | 99 | 99 | 99 |
| Benzene |  |  | -0.0408 | 0.154 | 0.286 | -0.0947 | 0.236 | -0.127 | 0.121 | 0.113 | 0.349 | 0.369 | 0.106 | 0.318 |
|  |  |  | 0.688 | 0.128 | 0.00423 | 0.35 | 0.019 | 0.21 | 0.232 | 0.265 | 0.000421 | 0.000189 | 0.294 | 0.00141 |
|  |  |  | 99 | 99 | 99 | 99 | 99 | 99 | 99 | 99 | 99 | 99 | 99 | 99 |
| Carbon Tetrachloride |  |  |  | -0.089 | -0.102 | -0.0562 | 0.206 | 0.0647 | -0.129 | 0.0913 | -0.115 | -0.137 | 0.522 | 0.227 |
|  |  |  |  | 0.38 | 0.314 | 0.58 | 0.0408 | 0.524 | 0.204 | 0.368 | 0.255 | 0.177 | 0.0000000402 | 0.0243 |
|  |  |  |  | 99 | 99 | 99 | 99 | 99 | 99 | 99 | 99 | 99 | 99 | 99 |
| Chloromethane |  |  |  |  | 0.714 | 0.237 | 0.466 | 0.357 | 0.326 | 0.0638 | 0.438 | 0.308 | 0.263 | -0.0365 |
|  |  |  |  |  | 0.0000002 | 0.0181 | 0.00000145 | 0.000308 | 0.00105 | 0.529 | 0.0000069 | 0.00198 | 0.00865 | 0.72 |
|  |  |  |  |  | 99 | 99 | 99 | 99 | 99 | 99 | 99 | 99 | 99 | 99 |
| Dichlorodifluoromethane |  |  |  |  |  | 0.154 | 0.485 | 0.243 | 0.355 | 0.0641 | 0.534 | 0.313 | 0.323 | 0.0271 |
|  |  |  |  |  |  | 0.128 | 0.000000461 | 0.0157 | 0.000337 | 0.528 | 0.0000000158 | 0.00169 | 0.00117 | 0.79 |
|  |  |  |  |  |  | 99 | 99 | 99 | 99 | 99 | 99 | 99 | 99 | 99 |
| Ethanol |  |  |  |  |  |  | 0.141 | 0.526 | 0.173 | 0.423 | 0.266 | 0.424 | -0.0307 | 0.0799 |
|  |  |  |  |  |  |  | 0.162 | 0.0000000302 | 0.0862 | 0.0000153 | 0.00793 | 0.0000143 | 0.762 | 0.431 |
|  |  |  |  |  |  |  | 99 | 99 | 99 | 99 | 99 | 99 | 99 | 99 |
| Freon 113 |  |  |  |  |  |  |  | 0.152 | 0.23 | -0.0373 | 0.2 | 0.133 | 0.497 | 0.0724 |
|  |  |  |  |  |  |  |  | 0.133 | 0.0224 | 0.713 | 0.0469 | 0.19 | 0.000000221 | 0.476 |
|  |  |  |  |  |  |  |  | 99 | 99 | 99 | 99 | 99 | 99 | 99 |
| Methanol |  |  |  |  |  |  |  |  | 0.185 | 0.436 | 0.351 | 0.509 | 0.177 | 0.277 |
|  |  |  |  |  |  |  |  |  | 0.0668 | 0.0000078 | 0.000392 | 0.0000001 | 0.0794 | 0.00567 |
|  |  |  |  |  |  |  |  |  | 99 | 99 | 99 | 99 | 99 | 99 |
| Methylene Chloride |  |  |  |  |  |  |  |  |  | 0.192 | 0.406 | 0.272 | 0.0697 | 0.133 |
|  |  |  |  |  |  |  |  |  |  | 0.0576 | 0.0000344 | 0.00665 | 0.492 | 0.188 |
|  |  |  |  |  |  |  |  |  |  | 99 | 99 | 99 | 99 | 99 |
| n-Hexane |  |  |  |  |  |  |  |  |  |  | 0.66 | 0.559 | -0.0113 | 0.366 |
|  |  |  |  |  |  |  |  |  |  |  | 0.0000002 | 0.00000000128 | 0.911 | 0.000211 |
|  |  |  |  |  |  |  |  |  |  |  | 99 | 99 | 99 | 99 |
| Propylene |  |  |  |  |  |  |  |  |  |  |  | 0.516 | 0.135 | 0.198 |
|  |  |  |  |  |  |  |  |  |  |  |  | 0.000000059 | 0.182 | 0.0501 |
|  |  |  |  |  |  |  |  |  |  |  |  | 99 | 99 | 99 |
| Toluene |  |  |  |  |  |  |  |  |  |  |  |  | -0.0128 | 0.278 |
|  |  |  |  |  |  |  |  |  |  |  |  |  | 0.9 | 0.0054 |
|  |  |  |  |  |  |  |  |  |  |  |  |  | 99 | 99 |
| Trichlorofluoromethane |  |  |  |  |  |  |  |  |  |  |  |  |  | 0.185 |
|  |  |  |  |  |  |  |  |  |  |  |  |  |  | 0.0667 |
|  |  |  |  |  |  |  |  |  |  |  |  |  |  | 99 |

Notes:

Cell contents are Spearman rank correlation coefficients (r_s_), p-values, and number of samples.

**Table S.3b Summary of Spearman Rank Correlation Results for 24-hour Measurement Data by Monitoring Site – Site 2**

|  | **Acetone** | **Benzene** | **Carbon  Tetrachloride** | **Chloromethane** | **Dichlorodifluoromethane** | **Ethanol** | **Freon 113** | **Methanol** | **Methylene  Chloride** | **n-Hexane** | **Propylene** | **Toluene** | **Trichlorofluoromethane** |
| --- | --- | --- | --- | --- | --- | --- | --- | --- | --- | --- | --- | --- | --- |
| 2-Butanone | 0.815 | 0.0358 | 0.126 | 0.195 | -0.0286 | 0.449 | -0.105 | 0.631 | 0.385 | 0.271 | 0.254 | 0.46 | 0.0598 |
|  | 0.0000002 | 0.709 | 0.188 | 0.0402 | 0.765 | 0.000000962 | 0.27 | 0.0000002 | 0.0000332 | 0.00414 | 0.00731 | 0.00000048 | 0.532 |
|  | 111 | 111 | 111 | 111 | 111 | 111 | 111 | 111 | 111 | 111 | 111 | 111 | 111 |
| Acetone |  | -0.0116 | 0.0988 | 0.168 | -0.0927 | 0.739 | -0.155 | 0.756 | 0.626 | 0.348 | 0.232 | 0.647 | 0.00324 |
|  |  | 0.903 | 0.302 | 0.0781 | 0.333 | 0.0000002 | 0.105 | 0.0000002 | 0.0000002 | 0.000193 | 0.0143 | 0.0000002 | 0.973 |
|  |  | 111 | 111 | 111 | 111 | 111 | 111 | 111 | 111 | 111 | 111 | 111 | 111 |
| Benzene |  |  | 0.0271 | 0.174 | 0.252 | 0.00876 | 0.23 | 0.0209 | 0.0506 | 0.406 | 0.35 | 0.437 | 0.288 |
|  |  |  | 0.778 | 0.0673 | 0.00778 | 0.927 | 0.0155 | 0.828 | 0.597 | 0.0000116 | 0.000177 | 0.00000197 | 0.00222 |
|  |  |  | 111 | 111 | 111 | 111 | 111 | 111 | 111 | 111 | 111 | 111 | 111 |
| Carbon Tetrachloride |  |  |  | -0.00585 | 0.0029 | 0.104 | 0.307 | 0.092 | 0.11 | 0.235 | -0.0577 | 0.105 | 0.412 |
|  |  |  |  | 0.951 | 0.976 | 0.277 | 0.00108 | 0.336 | 0.249 | 0.0133 | 0.547 | 0.272 | 0.00000834 |
|  |  |  |  | 111 | 111 | 111 | 111 | 111 | 111 | 111 | 111 | 111 | 111 |
| Chloromethane |  |  |  |  | 0.718 | -0.0692 | 0.364 | 0.245 | -0.12 | 0.0113 | 0.32 | -0.0417 | 0.312 |
|  |  |  |  |  | 0.0000002 | 0.47 | 0.0000966 | 0.00973 | 0.211 | 0.906 | 0.000663 | 0.663 | 0.000885 |
|  |  |  |  |  | 111 | 111 | 111 | 111 | 111 | 111 | 111 | 111 | 111 |
| Dichlorodifluoromethane |  |  |  |  |  | -0.249 | 0.562 | 0.0365 | -0.261 | -0.00436 | 0.304 | -0.124 | 0.493 |
|  |  |  |  |  |  | 0.00845 | 0.0000002 | 0.703 | 0.00569 | 0.964 | 0.00121 | 0.195 | 0.0000000505 |
|  |  |  |  |  |  | 111 | 111 | 111 | 111 | 111 | 111 | 111 | 111 |
| Ethanol |  |  |  |  |  |  | -0.164 | 0.636 | 0.742 | 0.358 | 0.145 | 0.674 | -0.107 |
|  |  |  |  |  |  |  | 0.0862 | 0.0000002 | 0.0000002 | 0.000125 | 0.129 | 0.0000002 | 0.261 |
|  |  |  |  |  |  |  | 111 | 111 | 111 | 111 | 111 | 111 | 111 |
| Freon 113 |  |  |  |  |  |  |  | -0.0913 | -0.186 | 0.00612 | 0.0591 | -0.129 | 0.602 |
|  |  |  |  |  |  |  |  | 0.34 | 0.0511 | 0.949 | 0.537 | 0.178 | 0.0000002 |
|  |  |  |  |  |  |  |  | 111 | 111 | 111 | 111 | 111 | 111 |
| Methanol |  |  |  |  |  |  |  |  | 0.451 | 0.266 | 0.197 | 0.583 | 0.134 |
|  |  |  |  |  |  |  |  |  | 0.000000838 | 0.00487 | 0.0386 | 0.0000002 | 0.161 |
|  |  |  |  |  |  |  |  |  | 111 | 111 | 111 | 111 | 111 |
| Methylene Chloride |  |  |  |  |  |  |  |  |  | 0.409 | 0.134 | 0.637 | -0.0472 |
|  |  |  |  |  |  |  |  |  |  | 0.00000963 | 0.162 | 0.0000002 | 0.623 |
|  |  |  |  |  |  |  |  |  |  | 111 | 111 | 111 | 111 |
| n-Hexane |  |  |  |  |  |  |  |  |  |  | 0.672 | 0.568 | 0.151 |
|  |  |  |  |  |  |  |  |  |  |  | 0.0000002 | 0.0000002 | 0.114 |
|  |  |  |  |  |  |  |  |  |  |  | 111 | 111 | 111 |
| Propylene |  |  |  |  |  |  |  |  |  |  |  | 0.254 | 0.14 |
|  |  |  |  |  |  |  |  |  |  |  |  | 0.00715 | 0.143 |
|  |  |  |  |  |  |  |  |  |  |  |  | 111 | 111 |
| Toluene |  |  |  |  |  |  |  |  |  |  |  |  | 0.055 |
|  |  |  |  |  |  |  |  |  |  |  |  |  | 0.566 |
|  |  |  |  |  |  |  |  |  |  |  |  |  | 111 |

Notes:

Cell contents are Spearman rank correlation coefficients (r_s_), p-values, and number of samples.

**Table S.3c Summary of Spearman Rank Correlation Results for 24-hour Measurement Data by Monitoring Site – Site 3**

|  | **Acetone** | **Benzene** | **Carbon  Tetrachloride** | **Chloromethane** | **Dichlorodifluoromethane** | **Ethanol** | **Freon 113** | **Methanol** | **Methylene  Chloride** | **n-Hexane** | **Propylene** | **Toluene** | **Trichlorofluoromethane** |
| --- | --- | --- | --- | --- | --- | --- | --- | --- | --- | --- | --- | --- | --- |
| 2-Butanone | 0.82 | -0.0716 | 0.118 | 0.271 | -0.0178 | 0.46 | -0.0989 | 0.638 | 0.355 | 0.303 | 0.319 | 0.387 | -0.0121 |
|  | 0.0000002 | 0.456 | 0.221 | 0.0043 | 0.853 | 0.000000563 | 0.304 | 0.0000002 | 0.000156 | 0.00134 | 0.00071 | 0.0000341 | 0.9 |
|  | 110 | 110 | 110 | 110 | 110 | 110 | 110 | 110 | 110 | 110 | 110 | 110 | 110 |
| Acetone |  | -0.069 | 0.122 | 0.278 | -0.0591 | 0.678 | -0.0447 | 0.702 | 0.576 | 0.331 | 0.28 | 0.557 | -0.001 |
|  |  | 0.473 | 0.204 | 0.0034 | 0.539 | 0.0000002 | 0.642 | 0.0000002 | 0.0000002 | 0.000444 | 0.00311 | 0.0000002 | 0.992 |
|  |  | 110 | 110 | 110 | 110 | 110 | 110 | 110 | 110 | 110 | 110 | 110 | 110 |
| Benzene |  |  | -0.0251 | 0.389 | 0.322 | 0.143 | 0.286 | 0.0491 | 0.0918 | 0.308 | 0.385 | 0.405 | 0.173 |
|  |  |  | 0.794 | 0.0000302 | 0.000629 | 0.136 | 0.00251 | 0.61 | 0.34 | 0.00109 | 0.0000368 | 0.0000134 | 0.0702 |
|  |  |  | 110 | 110 | 110 | 110 | 110 | 110 | 110 | 110 | 110 | 110 | 110 |
| Carbon Tetrachloride |  |  |  | -0.103 | -0.126 | 0.116 | 0.189 | 0.0724 | 0.162 | 0.269 | -0.0743 | 0.17 | 0.421 |
|  |  |  |  | 0.284 | 0.191 | 0.226 | 0.0476 | 0.452 | 0.0915 | 0.00457 | 0.44 | 0.076 | 0.00000539 |
|  |  |  |  | 110 | 110 | 110 | 110 | 110 | 110 | 110 | 110 | 110 | 110 |
| Chloromethane |  |  |  |  | 0.693 | 0.267 | 0.37 | 0.373 | 0.276 | 0.0454 | 0.42 | 0.177 | 0.278 |
|  |  |  |  |  | 0.0000002 | 0.00493 | 0.0000757 | 0.0000656 | 0.00359 | 0.637 | 0.00000585 | 0.0651 | 0.00338 |
|  |  |  |  |  | 110 | 110 | 110 | 110 | 110 | 110 | 110 | 110 | 110 |
| Dichlorodifluoromethane |  |  |  |  |  | 0.0316 | 0.502 | 0.103 | 0.0849 | -0.0525 | 0.394 | 0.0489 | 0.379 |
|  |  |  |  |  |  | 0.743 | 0.0000000301 | 0.282 | 0.377 | 0.586 | 0.0000234 | 0.611 | 0.0000488 |
|  |  |  |  |  |  | 110 | 110 | 110 | 110 | 110 | 110 | 110 | 110 |
| Ethanol |  |  |  |  |  |  | 0.0379 | 0.669 | 0.617 | 0.29 | 0.206 | 0.679 | 0.0906 |
|  |  |  |  |  |  |  | 0.694 | 0.0000002 | 0.0000002 | 0.00219 | 0.0313 | 0.0000002 | 0.346 |
|  |  |  |  |  |  |  | 110 | 110 | 110 | 110 | 110 | 110 | 110 |
| Freon 113 |  |  |  |  |  |  |  | -0.0531 | 0.181 | 0.0352 | 0.164 | 0.0973 | 0.497 |
|  |  |  |  |  |  |  |  | 0.581 | 0.0583 | 0.714 | 0.0859 | 0.311 | 0.0000000461 |
|  |  |  |  |  |  |  |  | 110 | 110 | 110 | 110 | 110 | 110 |
| Methanol |  |  |  |  |  |  |  |  | 0.495 | 0.152 | 0.173 | 0.467 | 0.0629 |
|  |  |  |  |  |  |  |  |  | 0.0000000499 | 0.112 | 0.0699 | 0.000000354 | 0.513 |
|  |  |  |  |  |  |  |  |  | 110 | 110 | 110 | 110 | 110 |
| Methylene Chloride |  |  |  |  |  |  |  |  |  | 0.256 | 0.104 | 0.556 | 0.215 |
|  |  |  |  |  |  |  |  |  |  | 0.00716 | 0.279 | 0.0000002 | 0.0241 |
|  |  |  |  |  |  |  |  |  |  | 110 | 110 | 110 | 110 |
| n-Hexane |  |  |  |  |  |  |  |  |  |  | 0.587 | 0.596 | 0.0243 |
|  |  |  |  |  |  |  |  |  |  |  | 0.0000002 | 0.0000002 | 0.8 |
|  |  |  |  |  |  |  |  |  |  |  | 110 | 110 | 110 |
| Propylene |  |  |  |  |  |  |  |  |  |  |  | 0.39 | 0.000201 |
|  |  |  |  |  |  |  |  |  |  |  |  | 0.0000287 | 0.998 |
|  |  |  |  |  |  |  |  |  |  |  |  | 110 | 110 |
| Toluene |  |  |  |  |  |  |  |  |  |  |  |  | 0.135 |
|  |  |  |  |  |  |  |  |  |  |  |  |  | 0.159 |
|  |  |  |  |  |  |  |  |  |  |  |  |  | 110 |

Notes:

Cell contents are Spearman rank correlation coefficients (r_s_), p-values, and number of samples.

**Table S.4 Comparison of Monitoring Site 1 VOC Measurements Based on Wind Direction^1,2^**

| **Compound** | **Days When the Winds Blew Predominantly from the S or SE^3^** | | | | **Days When the Winds Blew Predominantly from Other Directions^4^** | | | |
| --- | --- | --- | --- | --- | --- | --- | --- | --- |
|  | **Median Conc. (ppb)** | **Mean Conc. (ppb)** | **St. Dev. Conc. (ppb)** | **Max Conc. (ppb)** | **Median Conc. (ppb)** | **Mean Conc. (ppb)** | **St. Dev. Conc. (ppb)** | **Max Conc. (ppb)** |
| 2-Butanone | 0.42 | 0.51 | 0.32 | 1.58 | 0.30 | 0.42 | 0.34 | 1.93 |
| Acetone | 3.30 | 5.25 | 5.90 | 33.90 | 2.22 | 6.35 | 25.31 | 212.00 |
| Benzene | 0.12 | 0.13 | 0.05 | 0.26 | 0.13 | 0.13 | 0.06 | 0.29 |
| Carbon tetrachloride | 0.08 | 0.09 | 0.01 | 0.11 | 0.08 | 0.08 | 0.01 | 0.11 |
| Chloromethane | 0.52 | 0.52 | 0.15 | 1.10 | 0.55 | 0.57 | 0.19 | 1.62 |
| Dichlorodifluoromethane | 0.44 | 0.41 | 0.10 | 0.61 | 0.45 | 0.41 | 0.10 | 0.63 |
| Ethanol | 1.98 | 2.46 | 1.30 | 6.08 | 1.48 | 2.07 | 1.85 | 9.86 |
| Freon 113 | 0.07 | 0.07 | 0.02 | 0.09 | 0.07 | 0.07 | 0.01 | 0.09 |
| Methanol | 6.31 | 9.65 | 9.62 | 45.60 | 4.68 | 7.92 | 11.60 | 82.20 |
| Methylene chloride | 0.11 | 1.06 | 5.05 | 27.80 | 0.11 | 0.58 | 3.63 | 30.30 |
| n-Hexane | 0.21 | 0.27 | 0.30 | 1.76 | 0.12 | 0.21 | 0.35 | 2.37 |
| Propylene | 0.70 | 1.52 | 2.83 | 14.30 | 0.52 | 1.08 | 2.03 | 12.20 |
| Toluene | 0.13 | 0.97 | 4.39 | 24.20 | 0.11 | 1.12 | 8.26 | 68.70 |
| Trichlorofluoromethane | 0.24 | 0.25 | 0.04 | 0.33 | 0.23 | 0.24 | 0.03 | 0.33 |

Notes:

Conc. = Concentration; ppb = Parts Per Billion; St. Dev. = Standard Deviation; VOC = Volatile Organic Compound.

(1) Includes only VOCs detected in at least 75% of samples.

(2) Half of the detection limit was substituted for non-detects.

(3) Based on average daily wind directions in the southerly or southeasterly direction.

(4) Based on average daily wind directions in all wind directions other than southerly and southeasterly directions.

**Table S.5 Comparison of Monitoring Site 2 VOC Measurements Based on Wind Direction^1,2^**

| **Compound** | **Days When the Winds Blew Predominantly from the S or SE^3^** | | | | **Days When the Winds Blew Predominantly from Other Directions^4^** | | | |
| --- | --- | --- | --- | --- | --- | --- | --- | --- |
|  | **Median Conc. (ppb)** | **Mean Conc. (ppb)** | **St. Dev. Conc. (ppb)** | **Max Conc. (ppb)** | **Median Conc. (ppb)** | **Mean Conc. (ppb)** | **St. Dev. Conc. (ppb)** | **Max Conc. (ppb)** |
| 2-Butanone | 0.51 | 0.64 | 0.39 | 1.85 | 0.39 | 0.49 | 0.38 | 2.08 |
| Acetone | 4.99 | 6.30 | 5.14 | 27.70 | 3.69 | 5.06 | 4.17 | 21.80 |
| Benzene | 0.15 | 0.17 | 0.07 | 0.37 | 0.13 | 0.13 | 0.05 | 0.33 |
| Carbon tetrachloride | 0.08 | 0.09 | 0.02 | 0.21 | 0.08 | 0.09 | 0.02 | 0.21 |
| Chloromethane | 0.54 | 0.55 | 0.14 | 0.89 | 0.54 | 0.59 | 0.25 | 2.21 |
| Dichlorodifluoromethane | 0.44 | 0.42 | 0.12 | 0.65 | 0.44 | 0.42 | 0.12 | 0.93 |
| Ethanol | 4.30 | 27.57 | 77.46 | 442.00 | 2.39 | 10.93 | 16.74 | 87.70 |
| Freon 113 | 0.07 | 0.07 | 0.01 | 0.14 | 0.07 | 0.07 | 0.02 | 0.13 |
| Methanol | 9.04 | 13.55 | 13.25 | 54.80 | 8.02 | 10.23 | 10.04 | 51.60 |
| Methylene chloride | 0.35 | 1.86 | 4.78 | 27.40 | 0.22 | 0.82 | 1.19 | 5.21 |
| n-Hexane | 0.22 | 0.42 | 0.72 | 4.28 | 0.13 | 0.19 | 0.24 | 1.69 |
| Propylene | 0.92 | 1.46 | 2.83 | 18.00 | 0.61 | 0.95 | 1.53 | 9.87 |
| Toluene | 0.22 | 0.38 | 0.67 | 4.27 | 0.14 | 0.18 | 0.24 | 1.96 |
| Trichlorofluoromethane | 0.24 | 0.27 | 0.10 | 0.58 | 0.24 | 0.25 | 0.04 | 0.45 |

Notes:

Conc. = Concentration; ppb = Parts Per Billion; St. Dev. = Standard Deviation; VOC = Volatile Organic Compound.

(1) Includes only VOCs detected in at least 75% of samples.

(2) Half of the detection limit was substituted for non-detects.

(3) Based on average daily wind directions in the southerly or southeasterly direction.

(4) Based on average daily wind directions in all wind directions other than southerly and southeasterly directions.

**Table S.6 Comparison of VOC Data for Study Monitoring Sites with Data for the PADEP Background Florence Site**

| **Compound** | **% Detections** | | | | **Mean Conc. (ppb)** | | | | **Max. Detected Conc. (ppb)** | | | |
| --- | --- | --- | --- | --- | --- | --- | --- | --- | --- | --- | --- | --- |
|  | **Florence^a^** | **Study Monitoring Sites** | | | **Florence^a,b^** | **Study Monitoring Sites** | | | **Florence^a^** | **Study Monitoring Sites** | | |
|  |  | **#1** | **#2** | **#3** |  | **#1^b^** | **#2^b^** | **#3^b^** |  | **#1** | **#2** | **#3** |
| 1,1,1-Trichloroethane | 0% | 0% | 0% | 0% | ND | ND | ND | ND | ND | ND | ND | ND |
| 1,1,2,2-Tetrachloroethane | 0% | 0% | 0% | 0% | ND | ND | ND | ND | ND | ND | ND | ND |
| 1,1,2-Trichloroethane | 0% | 1% | 0% | 1% | ND | 0.03 | ND | 0.03 | ND | 0.07 | ND | 0.12 |
| 1,1-Dichloroethane | 0% | 0% | 0% | 0% | ND | ND | ND | ND | ND | ND | ND | ND |
| 1,1-Dichloroethylene | 0% | 0% | 0% | 0% | ND | ND | ND | ND | ND | ND | ND | ND |
| 1,2,4-Trichlorobenzene | 0% | 2% | 3% | 3% | ND | 0.03 | 0.03 | 0.03 | ND | 0.15 | 0.10 | 0.12 |
| 1,2,4-Trimethylbenzene | 0% | 7% | 11% | 7% | ND | 0.08 | 0.04 | 0.04 | ND | 2.71 | 0.40 | 0.46 |
| 1,2-Dibromoethane | 0% | 0% | 0% | 0% | ND | ND | ND | ND | ND | ND | ND | ND |
| 1,2-Dichloroethane | 2% | 3% | 3% | 4% | 0.02 | 0.03 | 0.03 | 0.04 | 0.03 | 0.12 | 0.17 | 0.57 |
| 1,2-Dichloropropane | 0% | 0% | 0% | 1% | ND | ND | ND | 0.03 | ND | ND | ND | 0.12 |
| 1,3,5-Trimethylbenzene | 0% | 3% | 4% | 3% | ND | 0.04 | 0.03 | 0.03 | ND | 0.79 | 0.14 | 0.24 |
| 1,3-Butadiene | 0% | 2% | 1% | 7% | ND | 0.03 | 0.03 | 0.04 | ND | 0.06 | 0.12 | 0.26 |
| 1-Bromopropane | 0% | 1% | 1% | 0% | ND | 0.03 | 0.03 | ND | ND | 0.25 | 0.07 | ND |
| 2-Butanone | 97% | 96% | 98% | 97% | 0.38 | 0.45 | 0.54 | 0.61 | 1.07 | 1.93 | 2.08 | 5.77 |
| 2-Hexanone | 0% | 17% | 22% | 23% | ND | 0.04 | 0.05 | 0.06 | ND | 0.14 | 0.27 | 1.27 |
| Acetone | 100% | 96% | 98% | 97% | 4.06 | 6.01 | 5.40 | 5.86 | 12.31 | 212.00 | 27.70 | 43.50 |
| Benzene | 94% | 92% | 96% | 96% | 0.18 | 0.13 | 0.15 | 0.17 | 0.59 | 0.29 | 0.37 | 0.95 |
| Bromodichloromethane | 0% | 0% | 0% | 0% | ND | ND | ND | ND | ND | ND | ND | ND |
| Bromoform | 0% | 0% | 0% | 0% | ND | ND | ND | ND | ND | ND | ND | ND |
| Bromomethane | 0% | 0% | 1% | 1% | ND | ND | 0.03 | 0.03 | ND | ND | 0.07 | 0.07 |
| c-1,2-Dichloroethylene | 0% | 0% | 0% | 0% | ND | ND | ND | ND | ND | ND | ND | ND |
| c-1,3-Dichloropropene | 0% | 0% | 0% | 0% | ND | ND | ND | ND | ND | ND | ND | ND |
| Carbon Tetrachloride | 100% | 96% | 96% | 97% | 0.10 | 0.08 | 0.09 | 0.08 | ND | 0.11 | 0.21 | 0.13 |
| Chlorobenzene | 0% | 0% | 2% | 1% | ND | ND | 0.03 | 0.03 | ND | ND | 0.06 | 0.07 |
| Chloroethane | 0% | 9% | 11% | 10% | ND | 0.04 | 0.04 | 0.06 | ND | 0.21 | 0.55 | 1.37 |
| Chloroform | 9% | 3% | 4% | 4% | 0.03 | 0.03 | 0.07 | 0.04 | 0.02 | 0.25 | 3.21 | 0.57 |
| Chloromethane | 100% | 96% | 98% | 97% | 0.61 | 0.56 | 0.57 | 0.56 | 0.78 | 1.62 | 2.21 | 1.66 |
| Cyclohexane | 12% | 25% | 30% | 38% | 0.04 | 0.06 | 0.08 | 0.08 | 0.10 | 1.10 | 2.49 | 0.89 |
| Dibromochloromethane | 0% | 0% | 0% | 0% | ND | ND | ND | ND | ND | ND | ND | ND |
| Dichlorodifluoromethane | 100% | 96% | 97% | 96% | 0.59 | 0.41 | 0.43 | 0.42 | 0.71 | 0.63 | 0.93 | 0.68 |
| Ethanol | N/A | 92% | 96% | 94% | N/A | 2.19 | 16.27 | 73.42 | N/A | 9.86 | 442.00 | 5,900.00 |
| Ethylbenzene | 0% | 3% | 8% | 12% | ND | 0.04 | 0.04 | 0.04 | ND | 0.97 | 0.29 | 0.31 |
| Freon 113 | 97% | 89% | 93% | 91% | 0.09 | 0.07 | 0.07 | 0.07 | 0.09 | 0.09 | 0.14 | 0.10 |
| Freon 114 | 0% | 0% | 0% | 0% | ND | ND | ND | ND | ND | ND | ND | ND |
| Hexachloro-1,3-Butadiene | 0% | 1% | 0% | 1% | ND | 0.03 | ND | 0.03 | ND | 0.06 | ND | 0.08 |
| m-Dichlorobenzene | 0% | 3% | 3% | 2% | ND | 0.03 | 0.03 | 0.03 | ND | 0.09 | 0.15 | 0.17 |
| Methanol | N/A | 96% | 98% | 97% | N/A | 8.44 | 11.08 | 13.29 | N/A | 82.20 | 54.80 | 122.00 |
| Methyl t-Butylether | 0% | 0% | 0% | 1% | ND | ND | ND | 0.03 | ND | ND | ND | 0.13 |
| Methylene Chloride | 67% | 83% | 87% | 91% | 0.14 | 0.72 | 1.15 | 1.00 | 2.44 | 30.30 | 27.40 | 19.70 |
| Methylisobutylketone | 0% | 4% | 11% | 11% | ND | 0.03 | 0.04 | 0.04 | ND | 0.18 | 0.12 | 0.31 |
| n-Heptane | 15% | 61% | 67% | 71% | 0.02 | 0.11 | 0.12 | 0.14 | 0.07 | 1.03 | 1.32 | 2.47 |
| n-Hexane | 84% | 93% | 89% | 90% | 0.08 | 0.23 | 0.27 | 0.31 | 0.21 | 2.37 | 4.28 | 7.57 |
| o-Dichlorobenzene | 0% | 2% | 2% | 2% | ND | 0.03 | 0.03 | 0.03 | ND | 0.07 | 0.09 | 0.16 |
| o-Xylene | 0% | 6% | 11% | 12% | ND | 0.05 | 0.04 | 0.04 | ND | 1.14 | 0.33 | 0.32 |
| p-Dichlorobenzene | 0% | 2% | 2% | 2% | ND | 0.03 | 0.03 | 0.03 | ND | 0.13 | 0.15 | 0.16 |
| p-Ethyltoluene | 0% | 3% | 4% | 3% | ND | 0.04 | 0.04 | 0.03 | ND | 0.55 | 0.26 | 0.29 |
| Propylene | 98% | 95% | 96% | 96% | 0.96 | 1.21 | 1.12 | 1.33 | 2.34 | 14.30 | 18.00 | 32.40 |
| p-Xylene + m-Xylene | 2% | 25% | 31% | 35% | 0.04 | 0.09 | 0.08 | 0.08 | 0.08 | 2.40 | 0.72 | 0.74 |
| Styrene | 0% | 2% | 23% | 19% | ND | 0.04 | 0.07 | 0.06 | ND | 0.48 | 0.73 | 0.65 |
| t-1,2-Dichloroethylene | 0% | 0% | 0% | 0% | ND | ND | ND | ND | ND | ND | ND | ND |
| t-1,3-Dichloropropene | 0% | 0% | 0% | 0% | ND | ND | ND | ND | ND | ND | ND | ND |
| Tetrachloroethylene | 0% | 2% | 4% | 1% | ND | 0.03 | 0.03 | 0.03 | ND | 0.13 | 0.21 | 0.07 |
| Tetrahydrofuran | 0% | 9% | 12% | 14% | ND | 0.04 | 0.04 | 0.04 | ND | 0.30 | 0.50 | 0.40 |
| Toluene | 98% | 92% | 90% | 90% | 0.13 | 1.08 | 0.25 | 0.43 | 0.25 | 68.70 | 4.27 | 9.16 |
| Trichloroethylene | 0% | 1% | 1% | 0% | ND | 0.03 | 0.03 | ND | ND | 0.07 | 0.11 | ND |
| Trichlorofluoromethane | 100% | 96% | 98% | 97% | 0.27 | 0.24 | 0.26 | 0.25 | 0.33 | 0.33 | 0.58 | 0.38 |
| Vinyl Chloride | 0% | 0% | 1% | 0% | ND | ND | 0.03 | ND | ND | ND | 0.07 | ND |

Notes:

Conc. = Concentration; Max. = Maximum; PADEP = Pennsylvania Dept. of Environmental Protection; ppb = Parts Per Billion; VOC = Volatile Organic Compound.

N/A indicates that there were no measurements of the specified compound and ND indicates that the compound was not detected.

(a) Florence monitor data for measurements from 10/2012 to 12/2013 from PADEP (2018 218-8169). Pennsylvania Dept. of Environmental Protection (PADEP). Long-Term Ambient Air Monitoring Project: Marcellus Shale Gas Facilities. 2018 July. 139 p.

(b) Mean estimates assume one-half the detection limit for non-detects.

**Table S.7 Maximum 24-hour VOC Concentrations Compared to Acute Health-based Air Comparison Values (HBACVs)**

| **Compound** | **Acute HBACV (ppb)** | **HBACV Source** | **Maximum Measured Conc. (ppb)** | | |
| --- | --- | --- | --- | --- | --- |
|  |  |  |  |  |  |
|  |  |  | **Monitoring Site 1** | **Monitoring Site 2** | **Monitoring Site 3** |
| 1,1,1-Trichloroethane | 2,000 | ATSDR acute inhalation MRL | ND | ND | ND |
| 1,1,2,2-Tetrachloroethane | N/A | N/A | ND | ND | ND |
| 1,1,2-Trichloroethane | 0.4 | Chronic US EPA RfC multiplied by 10 | 0.0686 | ND | 0.123 |
| 1,1-Dichloroethane | N/A | N/A | ND | ND | ND |
| 1,1-Dichloroethylene | 505 | Chronic US EPA RfC multiplied by 10 | ND | ND | ND |
| 1,2,4-Trichlorobenzene | 2.7 | Chronic US EPA RfC multiplied by 10 | 0.147 | 0.101 | 0.123 |
| 1,2,4-Trimethylbenzene | 122 | Chronic US EPA RfC multiplied by 10 | 2.71 | 0.403 | 0.457 |
| 1,2-Dibromoethane | 11.7 | Chronic US EPA RfC multiplied by 10 | ND | ND | ND |
| 1,2-Dichloroethane | 17.3 | Chronic US EPA RfC multiplied by 10 | 0.122 | 0.165 | 0.566 |
| 1,2-Dichloropropane | 50 | ATSDR acute inhalation MRL | ND | ND | 0.115 |
| 1,3,5-Trimethylbenzene | 122 | Chronic US EPA RfC multiplied by 10 | 0.786 | 0.137 | 0.243 |
| 1,3-Butadiene | 298 | ORNL RAIS acute inhalation RfC | 0.0584 | 0.122 | 0.264 |
| 1-Bromopropane | 1,000 | ATSDR acute inhalation MRL | 0.249 | 0.0654 | ND |
| 2-Butanone | 16,956 | Chronic US EPA RfC multiplied by 10 | 1.93 | 2.08 | 5.77 |
| 2-Hexanone | 73.2 | Chronic US EPA RfC multiplied by 10 | 0.14 | 0.269 | 1.27 |
| Acetone | 26,000 | ATSDR acute inhalation MRL | 212 | 27.7 | 43.5 |
| Benzene | 9 | ATSDR acute inhalation MRL | 0.288 | 0.369 | 0.947 |
| Bromodichloromethane | N/A | N/A | ND | ND | ND |
| Bromoform | N/A | N/A | ND | ND | ND |
| Bromomethane | 50 | ATSDR acute inhalation MRL | ND | 0.0677 | 0.0697 |
| c-1,2-Dichloroethylene | N/A | N/A | ND | ND | ND |
| c-1,3-Dichloropropene | 44.1 | Chronic US EPA RfC multiplied by 10 | ND | ND | ND |
| Carbon Tetrachloride | 302 | ORNL RAIS acute inhalation RfC | 0.112 | 0.206 | 0.127 |
| Chlorobenzene | 109 | Chronic US EPA RfC multiplied by 10 | ND | 0.0637 | 0.0728 |
| Chloroethane | 15,000 | ATSDR acute inhalation MRL | 0.21 | 0.545 | 1.37 |
| Chloroform | 100 | ATSDR acute inhalation MRL | 0.25 | 3.21 | 0.574 |
| Chloromethane | 500 | ATSDR acute inhalation MRL | 1.62 | 2.21 | 1.66 |
| Cyclohexane | 17,431 | Chronic US EPA RfC multiplied by 10 | 1.1 | 2.49 | 0.885 |
| Dibromochloromethane | N/A | N/A | ND | ND | ND |
| Dichlorodifluoromethane | 202 | Chronic US EPA RfC multiplied by 10 | 0.629 | 0.932 | 0.679 |
| Ethanol^1^ | 1,000,000 | NIOSH REL | 9.86 | 442 | 5,900 |
| Ethylbenzene | 5,000 | ATSDR acute inhalation MRL | 0.965 | 0.288 | 0.311 |
| Freon 113 | 6,524 | Chronic US EPA RfC multiplied by 10 | 0.0886 | 0.136 | 0.0961 |
| Freon 114 | N/A | N/A | ND | ND | ND |
| Hexachloro-1,3-Butadiene | N/A | N/A | 0.0592 | ND | 0.0787 |
| m-Dichlorobenzene | N/A | N/A | 0.0929 | 0.152 | 0.165 |
| Methanol | 21,367 | ORNL RAIS acute inhalation RfC | 82.2 | 54.8 | 122 |
| Methyl t-Butylether | 2,000 | ATSDR acute inhalation MRL | ND | ND | 0.13 |
| Methylene Chloride | 600 | ATSDR acute inhalation MRL | 30.3 | 27.4 | 19.7 |
| Methylisobutylketone | 7,323 | Chronic US EPA RfC multiplied by 10 | 0.183 | 0.121 | 0.311 |
| n-Heptane | 976 | Chronic US EPA RfC multiplied by 10 | 1.03 | 1.32 | 2.47 |
| n-Hexane | 1,986 | Chronic US EPA RfC multiplied by 10 | 2.37 | 4.28 | 7.57 |
| o-Dichlorobenzene | 333 | Chronic US EPA RfC multiplied by 10 | 0.069 | 0.0918 | 0.157 |
| p-Dichlorobenzene | 2,000 | ATSDR acute inhalation MRL | 0.125 | 0.146 | 0.159 |
| p-Ethyltoluene | N/A | N/A | 0.545 | 0.263 | 0.29 |
| Propylene | 17,431 | Chronic US EPA RfC multiplied by 10 | 14.3 | 18 | 32.4 |
| Styrene | 5,000 | ATSDR acute inhalation MRL | 0.477 | 0.726 | 0.647 |
| t-1,2-Dichloroethylene | 200 | ATSDR acute inhalation MRL | ND | ND | ND |
| t-1,3-Dichloropropene | 44.1 | Chronic US EPA RfC multiplied by 10 | ND | ND | ND |
| Tetrachloroethylene | 6 | ATSDR acute inhalation MRL | 0.128 | 0.206 | 0.0736 |
| Tetrahydrofuran | 6,782 | Chronic US EPA RfC multiplied by 10 | 0.295 | 0.501 | 0.402 |
| Toluene | 2,000 | ATSDR acute inhalation MRL | 68.7 | 4.27 | 9.16 |
| Trichloroethylene | 3.7 | Chronic US EPA RfC multiplied by 10 | 0.0698 | 0.105 | ND |
| Trichlorofluoromethane | N/A | N/A | 0.333 | 0.581 | 0.38 |
| Vinyl Chloride | 500 | ATSDR acute inhalation MRL | ND | 0.0654 | ND |
| Xylenes^2^ | 2,000 | ATSDR acute inhalation MRL | 1.77 | 1.05 | 1.06 |

Notes:

ATSDR = Agency for Toxic Substances and Disease Registry; Conc. = Concentration; MRL = Minimal Risk Level; NIOSH = US National Institute for Occupational Safety and Health; ORNL = Oak Ridge National Laboratory; ppb = Parts Per Billion; RAIS = Risk Assessment Information System; REL = Recommended Exposure Limit; RfC = Reference Concentration; US EPA = United States Environmental Protection Agency; VOC = Volatile Organic Compound.

N/A indicates that there is not an ATSDR acute inhalation MRL, chronic US EPA RfC, or ORNL RAIS acute inhalation RfC for the compound. ND indicates that the compound was not detected at a concentration above the measurement detection limit.

If the benchmarks were available in units of mass per cubic meter, they were converted to ppb assuming sea level pressure (101325 Pa) and a temperature of 25° C.

(1) No ATSDR acute inhalation MRL or ORNL RAIS acute inhalation RfC is currently available for ethanol.  The value shown is the NIOSH time-weighted average REL for ethanol that is intended to be protective of worker health for up to a 10-hour workday during a 40-hour workweek over a working lifetime (note that the NIOSH REL for ethanol is equivalent to the US Occupational Safety and Health Administration [OSHA] permissible exposure limit [PEL] for ethanol, as well as the American Conference of Governmental Industrial Hygienists [ACGIH] 15-minute Threshold Limit Value-Short-term Exposure Limit [TLV-STEL] for ethanol).

(2) The xylenes measurements represent the sum of m-,p- xylenes and o-xylene measurements.

**Table S.8 95% UCLs of Mean VOC Concentrations Compared to Chronic Health-based Air Comparison Values (HBACVs)**

| **Compound** | **Chronic HBACV (ppb)** | **HBACV Source** | **95% UCL of Mean Conc. (ppb)^1^** | | | | | |
| --- | --- | --- | --- | --- | --- | --- | --- | --- |
|  |  |  | **Total** | | | **Production Phase Only** | | |
|  |  |  | **Monitoring Site 1** | **Monitoring Site 2** | **Monitoring Site 3** | **Monitoring Site 1** | **Monitoring Site 2** | **Monitoring Site 3** |
| 1,1,1-Trichloroethane | 916 | US EPA RfC | ND | ND | ND | ND | ND | ND |
| 1,1,2,2-Tetrachloroethane | 0.3 | US EPA IUR | ND | ND | ND | ND | ND | ND |
| 1,1,2-Trichloroethane | 0.04 | US EPA RfC | NC (0.03) | ND | NC (0.03) | ND | ND | ND |
| 1,1-Dichloroethane | 15.4 | US EPA IUR | ND | ND | ND | ND | ND | ND |
| 1,1-Dichloroethylene | 50.5 | US EPA RfC | ND | ND | ND | ND | ND | ND |
| 1,2,4-Trichlorobenzene | 0.3 | US EPA RfC | 0.06 | 0.06 | 0.06 | NC (0.03) | ND | ND |
| 1,2,4-Trimethylbenzene | 12.2 | US EPA RfC | 0.30 | 0.08 | 0.08 | NC (0.03) | 0.06 | ND |
| 1,2-Dibromoethane | 0.02 | US EPA IUR | ND | ND | ND | ND | ND | ND |
| 1,2-Dichloroethane | 1.0 | US EPA IUR | 0.06 | 0.06 | 0.08 | ND | 0.07 | NC (0.03) |
| 1,2-Dichloropropane | 0.9 | US EPA RfC | ND | ND | NC (0.03) | ND | ND | ND |
| 1,3,5-Trimethylbenzene | 12.2 | US EPA RfC | 0.08 | 0.06 | 0.07 | ND | ND | ND |
| 1,3-Butadiene | 0.9 | US EPA RfC | 0.06 | NC (0.03) | 0.07 | ND | ND | NC (0.03) |
| 1-Bromopropane | 19.9 | US EPA RfC | NC (0.03) | NC (0.03) | ND | NC (0.03) | NC (0.03) | ND |
| 2-Butanone | 1,696 | US EPA RfC | 0.51 | 0.60 | 0.68 | 0.42 | 0.53 | 0.53 |
| 2-Hexanone | 7.3 | US EPA RfC | 0.07 | 0.08 | 0.13 | 0.07 | 0.07 | 0.07 |
| Acetone | 13,050 | US EPA RfC | 5.13 | 6.09 | 6.69 | 3.15 | 6.88 | 4.79 |
| Benzene | 4.0 | US EPA IUR | 0.15 | 0.15 | 0.17 | 0.15 | 0.16 | 0.16 |
| Bromodichloromethane | 0.4 | US EPA IUR | ND | ND | ND | ND | ND | ND |
| Bromoform | 8.8 | US EPA IUR | ND | ND | ND | ND | ND | ND |
| Bromomethane | 1.3 | US EPA RfC | ND | NC (0.03) | NC (0.03) | ND | NC (0.03) | ND |
| c-1,2-Dichloroethylene | N/A | N/A | ND | ND | ND | ND | ND | ND |
| c-1,3-Dichloropropene | 4.4 | US EPA RfC | ND | ND | ND | ND | ND | ND |
| Carbon Tetrachloride | 2.7 | US EPA IUR | 0.09 | 0.09 | 0.09 | 0.09 | 0.09 | 0.09 |
| Chlorobenzene | 10.9 | US EPA RfC | ND | 0.06 | NC (0.03) | ND | ND | ND |
| Chloroethane | 3,790 | US EPA RfC | 0.07 | 0.07 | 0.08 | 0.06 | NC (0.03) | 0.06 |
| Chloroform | 0.9 | US EPA IUR | 0.07 | 0.15 | 0.08 | NC (0.03) | 0.06 | ND |
| Chloromethane | 43.6 | US EPA RfC | 0.59 | 0.61 | 0.60 | 0.51 | 0.55 | 0.51 |
| Cyclohexane | 1,743 | US EPA RfC | 0.13 | 0.20 | 0.16 | 0.07 | 0.30 | 0.09 |
| Dibromochloromethane | N/A | N/A | ND | ND | ND | ND | ND | ND |
| Dichlorodifluoromethane | 23.8 | US EPA RfC | 0.43 | 0.45 | 0.44 | 0.39 | 0.41 | 0.39 |
| Ethanol^2^ | 1,000,000 | NIOSH REL | 2.94 | 36.17 | 308.20 | 2.43 | 51.14 | 530.10 |
| Ethylbenzene | 9.2 | US EPA IUR | 0.09 | 0.07 | 0.08 | ND | 0.07 | 0.06 |
| Freon 113 | 652 | US EPA RfC | 0.07 | 0.08 | 0.07 | 0.07 | 0.08 | 0.07 |
| Freon 114 | N/A | N/A | ND | ND | ND | ND | ND | ND |
| Hexachloro-1,3-Butadiene | 0.4 | US EPA IUR | NC (0.03) | ND | NC (0.03) | ND | ND | ND |
| m-Dichlorobenzene | N/A | N/A | 0.06 | 0.06 | 0.07 | NC (0.03) | ND | ND |
| Methanol | 15,262 | US EPA RfC | 13.27 | 12.93 | 15.26 | 5.47 | 11.66 | 11.43 |
| Methyl t-Butylether | 107 | US EPA IUR | ND | ND | NC (0.03) | ND | ND | ND |
| Methylene Chloride | 173 | US EPA RfC | 2.52 | 2.39 | 2.08 | 0.12 | 2.32 | 1.58 |
| Methylisobutylketone | 732 | US EPA RfC | 0.07 | 0.07 | 0.08 | NC (0.03) | 0.07 | 0.06 |
| n-Heptane | 97.6 | US EPA RfC | 0.19 | 0.20 | 0.26 | 0.08 | 0.13 | 0.10 |
| n-Hexane | 199 | US EPA RfC | 0.38 | 0.47 | 0.29 | 0.19 | 0.40 | 0.24 |
| o-Dichlorobenzene | 33.3 | US EPA RfC | 0.06 | 0.06 | 0.07 | NC (0.03) | ND | ND |
| p-Dichlorobenzene | 1.5 | US EPA IUR | 0.06 | 0.06 | 0.06 | NC (0.03) | ND | ND |
| p-Ethyltoluene | N/A | N/A | 0.08 | 0.07 | 0.07 | ND | ND | ND |
| Propylene | 1,743 | US EPA RfC | 2.22 | 1.97 | 1.30 | 0.73 | 0.75 | 0.79 |
| Styrene | 235 | US EPA RfC | 0.09 | 0.11 | 0.12 | ND | 0.14 | 0.08 |
| t-1,2-Dichloroethylene | N/A | N/A | ND | ND | ND | ND | ND | ND |
| t-1,3-Dichloropropene | 4.4 | US EPA RfC | ND | ND | ND | ND | ND | ND |
| Tetrachloroethylene | 5.9 | US EPA RfC | 0.06 | 0.06 | NC (0.03) | ND | 0.07 | ND |
| Tetrahydrofuran | 678 | US EPA RfC | 0.07 | 0.09 | 0.07 | NC (0.03) | 0.07 | 0.06 |
| Toluene | 1,327 | US EPA RfC | 4.27 | 0.43 | 0.94 | 0.12 | 0.32 | 0.23 |
| Trichloroethylene | 0.4 | US EPA RfC | NC (0.03) | NC (0.03) | ND | ND | ND | ND |
| Trichlorofluoromethane | N/A | N/A | 0.25 | 0.27 | 0.25 | 0.24 | 0.27 | 0.25 |
| Vinyl Chloride | 8.9 | US EPA IUR | ND | NC (0.03) | ND | ND | ND | ND |
| Xylenes^3^ | 23.0 | US EPA RfC | 0.32 | 0.19 | 0.19 | 0.09 | 0.20 | 0.11 |

Notes:

Conc. = Concentration; IUR = Inhalation Unit Risk; NIOSH = US National Institute for Occupational Safety and Health; ppb = Parts Per Billion; REL = Recommended Exposure Limit; RfC = Reference Concentration; UCL = upper confidence limit; US EPA = United States Environmental Protection Agency; VOC = Volatile Organic Compound.

N/A indicates that there is not an US EPA RfC or IUR for the compound. ND indicates that the compound was not detected at concentration above the measurement detection limit. NC indicates that a 95% UCL was not calculated because there was just a single detected concentration; for these cases, mean concentrations are shown in parentheses where half of the detection limit was substituted for any measurements below the detection limit

The IURs were converted to units of μg/m3 assuming 1 in 10,000 risk. The IURs and RfCs were converted from units of mass per cubic meter to ppb assuming sea level pressure (101,325 Pa) and a temperature of 25° C.

(1) For VOCs detected at least twice, 95% UCLs of mean concentrations were calculated using US EPA's ProUCL software, with reporting of 95% UCLs for the methods recommended by the software.

(2) No US EPA RfC is currently available for ethanol.  The value shown is the NIOSH time-weighted average REL for ethanol that is intended to be protective of worker health for up to a 10-hour workday during a 40-hour workweek over a working lifetime (note that the NIOSH REL for ethanol is equivalent to the US Occupational Safety and Health Administration [OSHA] permissible exposure limit [PEL] for ethanol, as well as the American Conference of Governmental Industrial Hygienists [ACGIH] 15-minute Threshold Limit Value-Short-term Exposure Limit [TLV-STEL] for ethanol).

(3) The xylenes measurements at the three Yonker sites represent the sum of m-,p- xylenes and o-xylene measurements.


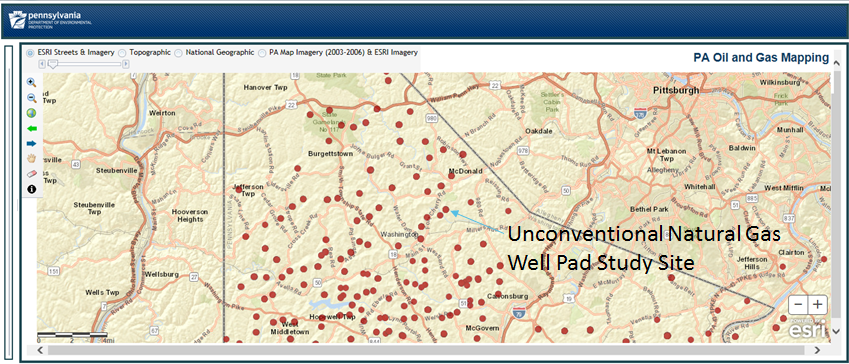


**Figure S.1 Location of the Unconventional Natural Gas Well Pad Study Site Relative to Other Area Active Unconventional Natural Gas Wells**. The location of the study site is indicated by a light blue arrow, and red circles indicate the locations of active unconventional natural gas well pad sites. [18]

**Figure S.2 Box Plots of Measured Hourly PM_2.5_ Concentrations by Study Well Pad Activity Period for Winds from Southerly and Southeasterly *versus* Other Wind Directions.** PM_2.5_ = Fine particulate matter less than 2.5 micrometers in diameter. (***) and (**) indicate that the distributions of hourly PM_2.5_ data were found to be statistically different (p<0.001 and p<0.01, respectively) between the two wind direction categories using the Mann-Whitney rank sum test, while (NS) indicates no statistical difference was found (p-value>0.05).
